# Synthesis of coumarin-containing poly(2-oxazoline)s and light-induced crosslinking for hydrogel formation

**DOI:** 10.1007/s00706-022-03013-8

**Published:** 2022-12-08

**Authors:** Carola Haslinger, Anna Zahoranová, Stefan Baudis

**Affiliations:** 1 Christian Doppler Laboratory for Advanced Polymers for Biomaterials and 3D Printing, Getreidemarkt 9, 1060 Vienna, Austria; 2 Institute of Applied Synthetic Chemistry, Technische Universität Wien, Getreidemarkt 9, 1060 Vienna, Austria

**Keywords:** Thermoresponsive behavior, Photochemistry, Polymerizations, Monomer synthesis, Kinetics

## Abstract

Herein, we present a new route to synthesize poly(2-oxazoline)s (POx) with coumarin moieties as pendant groups that can be crosslinked by irradiation to form hydrogels. The synthesis of a novel coumarin-containing 2-oxazoline monomer, 2-[(4-methyl-7-coumarinyloxy)methyl]oxazoline is described in four steps and further its subsequent homo- and copolymerization with 2-ethyl-2-oxazoline (EtOx) via cationic ring-opening polymerization (CROP). The received polymers with different coumarin content (8, 4 and 2 mol%) were compared with a known copolymer synthesized via postpolymerization modification of partially hydrolyzed poly(2-ethyl-2-oxazoline) (PEtOx) with a coumarin derivative. The thermoresponsive behavior of the POx in aqueous solutions was investigated, showing a clear difference in the cloud point temperature (*T*_*cp*_) between the polymers synthesized via different strategies. The hydrogel formation was achieved by irradiation with UV light by the following two methods: on the one hand with the pure coumarin-containing POx and on the other hand mixed with poly(ethylene glycol) diacrylate (PEGDA). The obtained hydrogels were compared regarding their swelling degree and their gel content, whereas the swelling degree in water of prepared hydrogels can be tuned by using POx by varying the coumarin content.

**Graphical abstract:**

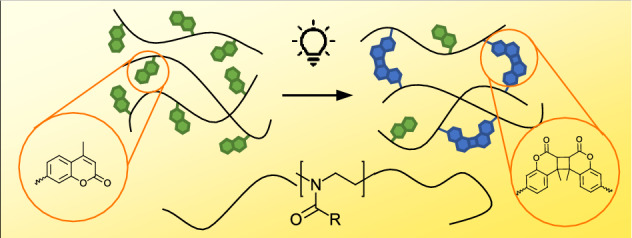

**Supplementary Information:**

The online version contains supplementary material available at 10.1007/s00706-022-03013-8.

## Introduction

Synthetic hydrogels have become a crucial component in the field of tissue engineering and drug delivery [1]. To prepare hydrogels from an aqueous solution of linear (pre)polymers, the (pre)polymers can be crosslinked either chemically, via covalent bonds, or physically, also known as non-covalent crosslinking. Non-covalent crosslinking includes hydrogen bonding, hydrophobic and ionic interactions [2].

One of the most convenient ways for chemical crosslinking is photocrosslinking, where the light is used to initiate the gelation process. The advantage of this method is the fast speed of the process, what enables the system to fully crosslink within a few minutes or even seconds. This property is a prerequisite for 3D printing of the material, which is sometimes even possible in the presence of living cells [3]. The main drawback for use of photocrosslinkable materials in biomedical field is the cytotoxicity of the most commonly used photoinitiators.

One method to overcome the drawback of the cytotoxicity of the initiator is to use initiator-free photocrosslinking methods, such as coumarin dimerization. Coumarin is a biomolecule that naturally occurs in tonka beans, it is also used in food industry. Coumarin is tested to be biocompatible in small doses (*LD50* = 293 mg kg^−1^, rat, oral) [4]. When irradiated with light above 360 nm, coumarin units form dimers. These dimers can be reversibly cleaved upon irradiation with UV light below 280 nm. The coumarin units can be incorporated into or grafted onto polymer chains, what enables the crosslinking of the polymers upon irradiation.

The advantages of using coumarin groups for photocrosslinking have been recognized by various research groups, which have employed them to synthesize hydrogels. Nagata et al. [5] synthesized poly(ethylene glycol) (PEG) with coumarin moieties via polycondensation. The PEG chains were further crosslinked after a short irradiation time within minutes. The swelling degree of the resulting hydrogels depended on the irradiation time and the molecular weight (MW) of the polymers, making them a promising substrate for drug delivery.

The introduction of coumarin groups via grafting coumarin derivatives onto partially hydrolyzed poly(2-alkyl-2-oxazoline)s was investigated by Chujo et al. [6]. Here, the swelling properties were also controlled by irradiation time and additionally by the content of coumarin in the polymer chains.

Kabb et al. [7] investigated the copolymerization of vinyl monomers containing coumarin functionalities with *N,N*-dimethylacrylamide (DMA). The reversibility of the polymers crosslinking was studied and further used for photopatterned hydrogels and 3D printing of hollow structures. Also here, the materials displayed low cytotoxicity, important for further biomedical applications.

In addition to versatile and biocompatible graft, the proper selection of polymer backbone is of a great importance. Among other hydrophilic polymers used for biomedical application, poly(2-oxazoline)s (POx) are a popular choice. POx are structural isomers of polypeptides. They have shown excellent biocompatibility in several in vitro [8] and in vivo tests [9, 10], what makes them promising for this application field. An advantage compared to the “gold standard” PEG is a still limited commercial use, resulting in no allergic reactions against POx being reported yet [11–13]. Numerous research groups are studying POx as drug or gene carriers [14], protein conjugates [15], or hydrogels for tissue engineering [6]. In addition to this academic interest, POx-drug conjugates have already been tested in clinical trials for use in human medicine [16, 17].

POx have been synthesized successfully since 1966 [18–21] via cationic ring-opening polymerization (CROP). If performed under appropriate anhydrous conditions, this polymerization exhibits living character, which is reflected in the well-controlled properties of the resulting polymer. The advantages of this polymerization are a narrow molar mass dispersity, a well-defined molar mass and a possibility of end group modification. Further, various functional groups can be easily introduced into the POx structure.

The first example of hydrogels crosslinked with coumarin moieties was actually based on POx, as introduced in 1989 by Chujo et al. [6]. Interestingly, since this first pioneering work, to the best of our knowledge only one further work on POx-based coumarin hydrogels followed. Nahm and co-workers [22] used POx-based material as biomaterial ink for the additive manufacturing of microperiodic hydrogel scaffolds. Additionally, the self-assembled POx nanoparticles end-capped with coumarin moieties were prepared by Korchia et al. [23].

In the work of Chujo et al. [6], coumarin-modified POx were prepared by multiple-step synthesis starting from hydrolyzed POx. In addition to laborious multiple-step synthesis with several purification steps, another disadvantage arises in the final step, where a coumarin derivative is added onto the hydrolyzed POx copolymer backbone. As it could be problematic to ensure a full conversion of this reaction, some poly(ethylene imine) (PEI) units could remain unmodified on the polymer backbone. As reported in literature [24], PEI is highly cytotoxic and is, therefore, mainly used in detergents and adhesives. Additionally, significant batch-to-batch variations in copolymer composition can be observed using this method.

In this article, we proposed an alternative method to obtain reproducible coumarin-containing POx without any PEI units in the backbone. First, a new 2-oxazoline monomer with a coumarin derivative as pendant group was synthesized. The homo- and copolymerization kinetics of the new monomer with hydrophilic MeOx and EtOx were determined. As a comparison, poly(2-ethyl-2-oxazoline) (PEtOx) was partially hydrolyzed and modified with coumarin derivatives, similar to the work of Chujo et al. [6]. Both copolymer types were compared in terms of their thermoresponsive behavior in water. To further exhibit the potential of the synthesized copolymers as hydrogel precursors, the copolymers were crosslinked via irradiation with and without poly(ethylene glycol) diacrylate (PEGDA). Finally, the swelling properties of the resulting hydrogels in water were studied.

## Results and discussion

### Monomer synthesis

To achieve a better control of the composition of resulting polymer chain, we proposed a method to prepare a poly(2-oxazoline) with coumarin moieties as pendant groups via copolymerization of a hydrophilic 2-oxazoline and a 2-oxazoline monomer containing pendant coumarin group.

In general, there exist several methods to synthesize 2-oxazoline monomers [25]. We have chosen a well-established method consisting of the synthesis of a chloroethyl-amide and its ring closing.

**Fig. 1:**
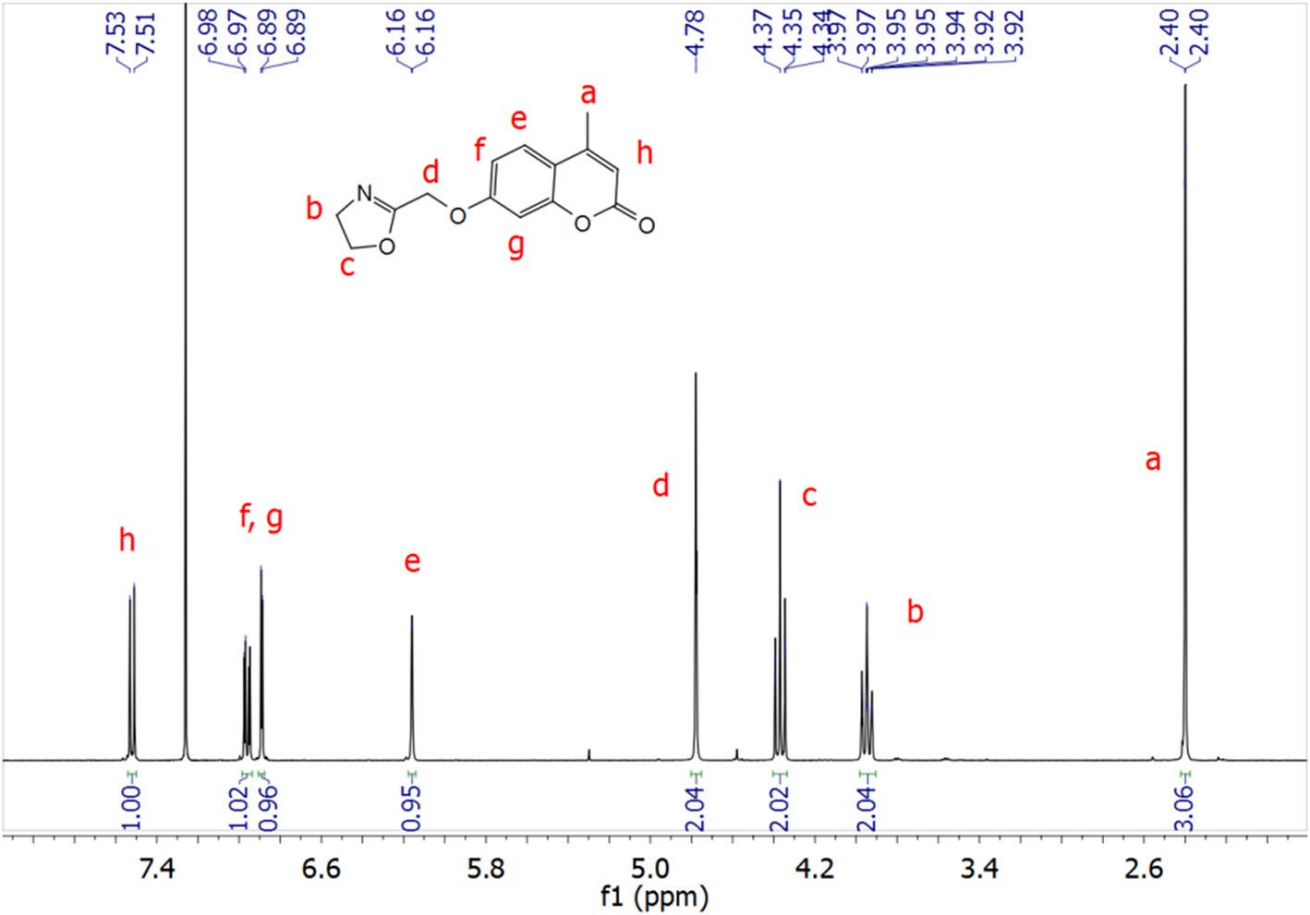
^1^H NMR spectrum of **CoumOx** in CDCl_3_

The novel monomer 2-[(4-methyl-7-coumarinyloxy)-methyl]oxazoline (**CoumOx**) was prepared in four steps according to Scheme 1 starting with the esterification of 4-methylumbelliferone. The first and second step to obtain **CoumOx** was performed according to Chujo et al. [6]. We started with the synthesis of (4-methylcoumarin-7-yloxy)-acetate (**1**) from 4-methylumbellicferone and ethyl bromoacetate yielding 69%. The ester group was hydrolyzed under basic conditions to result in (4-methylcoumarin-7-yloxy)acetic acid (**2**, 96% yield). Afterwards, *N*-2-chloroethyl(4-methyl-7-coumarinyloxy)acetamide (**3**) was synthesized similarly as described by Cesana et al. [26] for the synthesis of protected thiol-containing 2-oxazoline. Here, 2-chloroethylamide hydrochloride was used for amide formation by a mixed anhydride method with a final yield of 74%. The final step was the ring closing with anhydrous K_2_CO_3_ as base in dry methanol under elevated temperature [26] (45% yield). The successful synthesis of the novel 2-oxazoline monomer **CoumOx** was confirmed by ^1^H and ^13^C NMR spectroscopy (see Fig. 1 and Supporting Information, Fig. ESI 1), melting point (170.0–220.0 °C) and HRMS measurements (282.2798 g/mol).

**Figure:**
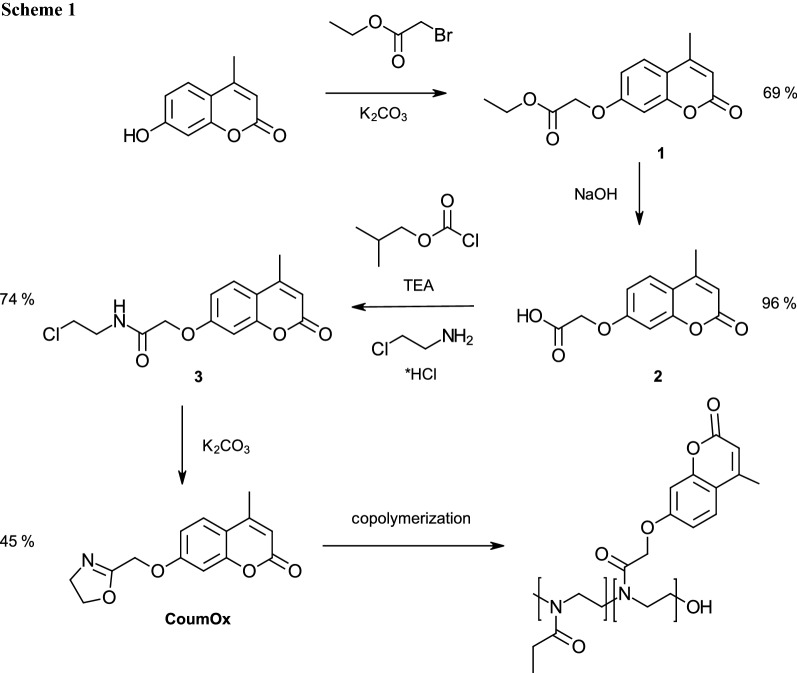

### Homopolymerization and copolymerization kinetics

As **CoumOx** is not known in literature, the rate of its homopolymerization was investigated before proceeding to copolymerize it with another 2-oxazoline. Using methyl triflate (MeOTf) as initiator (M:I 50:1), **CoumOx** was polymerized as a 0.2 M solution in anhydrous acetonitrile at 75 °C. This solvent was chosen based on its ability to dissolve a broad range of 2-oxazoline monomers, its low toxicity compared to halogenated solvents and its easy removal from the final product, as the boiling point is low and it can be removed via dialysis as it is water miscible. Therefore, homopolymerization experiments with 2-ethyl-2-oxazoline (EtOx) and 2-methyl-2-oxazoline (MeOx) and further copolymerization experiments were also conducted in acetonitrile to ensure predictable copolymerization kinetics. Unfortunately, performing experiments at higher monomer concentration of **CoumOx** was not possible in acetonitrile due to solubility issues (appearance of visible aggregates). It should be noted that although we did not study potential formation of aggregates not visible to naked eye, we believe that it would not disturb the polymerization, as it proceeds rapidly and until full conversion under the studied conditions.

Samples were withdrawn from the reaction mixture in selected time intervals to investigate the homopolymerization kinetics of **CoumOx** via ^1^H NMR spectroscopy. The sample was withdrawn from the solution and diluted with CDCl_3_ prior the polymerization mixture started to precipitate due to the decrease of the temperature.

**Fig. 2:**
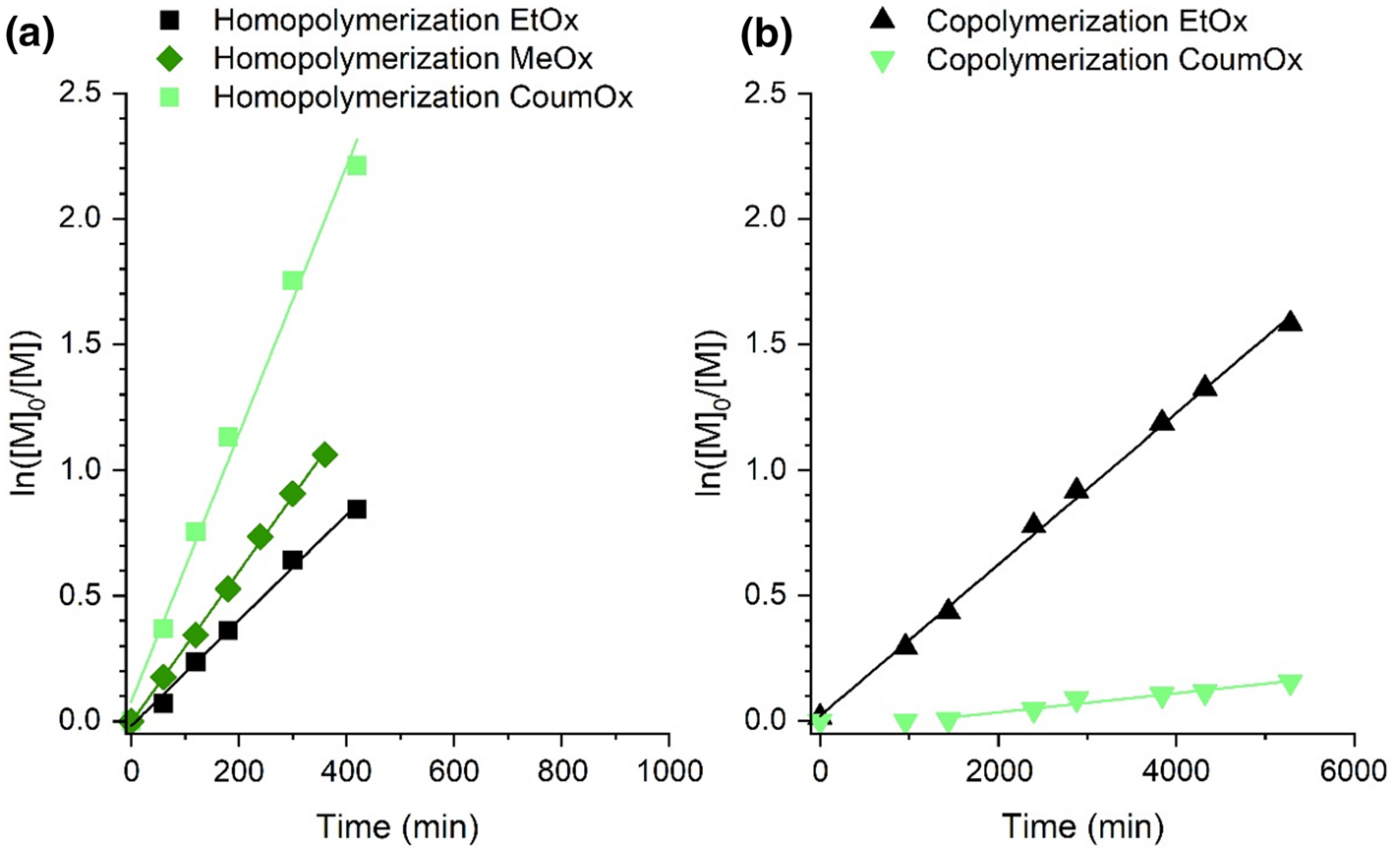
**a** Homopolymerization kinetics of **CoumOx** compared with EtOx and MeOx in acetonitrile as solvent (3 M for EtOx and MeOx, 0.2 M for **CoumOx**) and MeOTf as initiator (M:I 50:1) with a polymerization temperature of 75 °C, **b** kinetics of the copolymerization using EtOx and **CoumOx** (molar ratio 4:1) in acetonitrile (3 M) at 75 °C and MeOTf as initiator (M:I 50:1)

The conversion of the samples was determined using ^1^H NMR spectroscopy; the calculations are described in further details in the Supporting Information, chapter 2. The results were plotted in Fig. 2a, compared to the homopolymerization of EtOx and MeOx under similar polymerization conditions (in acetonitrile at 75 °C).

The only difference was the lower concentration of the monomer **CoumOx** (0.2 M in comparison to 3 M of EtOx and MeOx). All homopolymerization experiments for the determination of their kinetics were conducted over 6 to 7 h, and full conversion was reached after around 24 h in case of MeOx and EtOx, respectively. Therefore, the increase of the viscosity of the polymer mixture at high conversions was neglected.

All the homopolymerizations followed first-order kinetics, which manifested the living character of the polymerization. Surprisingly, **CoumOx** exhibited a faster apparent propagation rate constant (*k*_p_, 5.33 dm^3^ mol^−1^ s^−1^ for **CoumOx**) in comparison to EtOx and MeOx (2.10 × 10^–3^ dm^3^ mol^−1^ s^−1^ and 3.00 × 10^–3^ dm^3^ mol^−1^ s^−1^; see Table 1). As the concentration of **CoumOx** is lower than EtOx and MeOx, the difference in the *k*_p_ values under the same conditions are expected to be even more pronounced.

**Table 1:** Propagation rate constant (*k*_p_) values of the homo-and copolymerization kinetic experiments and their coefficient of determination (*R*^2^)

Experiment	*k*_p_/dm^3^ mol^−1^ s^−1^	*R*^2^
Homopolymerization EtOx	2.10 × 10^–3^	0.9931
Homopolymerization MeOx	3.00 × 10^–3^	0.9988
Homopolymerization **CoumOx**	5.33 × 10^–3^	0.9879
Copolymerization EtOx	3.01 × 10^–4^	0.9980
Copolymerization CoumOx	3.79 × 10^–5^	0.9515

For 2-oxazolines, *k*_p_ is affected by the polymerization conditions, such as temperature, solvent, concentration of the propagating species and the structure of 2-oxazoline monomer, i.e., the pendant group. The monomer structure influences the nucleophilicity of the imine functionality and also the positive charge at the 5-position of the oxazolinium species. Additionally, steric hindrance is also affecting *k*_p_ [27].

Bouten et al. [28] experienced a similar polymerization kinetics of 2-oxazolines containing methyl ester group. In that case, the monomer polymerized also faster than EtOx. This was explained by the interaction of the methyl ester group with the living chain end, which increased its electrophilicity. In our case, to elucidate the exact mechanism will require further more detailed examination, what is beyond the scope of the current paper.

The homopolymerization of **CoumOx** was terminated after full conversion. After the evaporation of the solvent of the reaction mixture, the polymer was received as white solid. The white solid could not be further redissolved in a wide range of solvents, including DMSO, H2O, CHCl_3_, DCM, DMF, dimethyl acetamide, EtOH, MeOH, ethyl acetate, acetone, THF, and 1,4-dioxane. Due to the variety of solvents, no more solubility experiments of the **CoumOx** homopolymer were performed.

A potential explanation is the crosslinking of the coumarin moieties although we tried to minimize the possible crosslinking by the execution of all experiments under yellow light with exclusion of wavelengths below 480 nm. Another reason could be the formation of inter- and intramolecular interactions once the solvent is removed, what would explain why the polymer is not soluble once the solvent was removed. In the current work, no further analytical investigations were conducted due to the insolubility of the polymer.

Further, we investigated the copolymerization kinetics of **CoumOx** with more hydrophilic EtOx monomer (molar ratio 1:4) in acetonitrile at 75 °C as shown in Fig. 2b. The *k*_p_ values of all kinetic experiments were calculated from the slope of the first-order kinetic plots and are listed in Table 1.

Similar *k*_p_ values for the homopolymerization of MeOx and EtOx in acetonitrile at 80 °C (25.1 ± 3.9 × 10^–4^ dm^3^ mol^−1^ s^−1^ and 16.2 ± 4.2 × 10^–4^ dm^3^ mol^−1^ s^−1^, respectively) were reported by Hoogenboom et al. [29].

The most pronounced change in Fig. 2b compared to Fig. 2a is the inversion of *k*_p_ of **CoumOx** and EtOx (3.79 × 10^–5^ dm^3^ mol^−1^ s^−1^ and 3.01 × 10^–4^ dm^3^ mol^−1^ s^−1^). Additionally, *k*_p_ of both **CoumOx** and EtOx (2.10 × 10^–3^ dm^3^ mol^−1^ s^−1^) is lower during the copolymerization than during their homopolymerization (3.79 × 10^–5^ dm^3^ mol^−1^ s^−1^ instead of 5.33 × 10^–3^ dm^3^ mol^−1^ s^−1^ for **CoumOx**; 3.01 × 10^–4^ dm^3^ mol^−1^ s^−1^ instead of 2.10 × 10^–3^ dm^3^ mol^−1^ s^−1^ for EtOx).

Based on these kinetics studies, the copolymerization of **CoumOx** and EtOx is presumably resulting in gradient copolymers. At the beginning of the reaction, EtOx is preferably consumed, while no conversion of **CoumOx** is observed for 1440 min. The monomer **CoumOx** is incorporated into the polymer chains at prolonged reaction times. The detailed calculation of the copolymerization parameters was beyond the scope of the current paper.

Being aware of the gradient structure of resulting copolymers, we were curious if also using these starting materials, the preparation of precursors for crosslinked hydrogels will be possible. To this end, a small series of gradient copolymers (**PEtOx_Coum2**, **PEtOx_Coum4**, **PEtOx_Coum8**, degree of polymerization (DP) 100) containing EtOx and differing amount of **CoumOx** was prepared under the same conditions (acetonitrile at 75 °C). We choose EtOx, as a less hydrophilic alternative to MeOx, to minimize the differences in polarity between the two used monomers. For **PEtOx_Coum2** and **PEtOx_Coum4**, the solid MeONs was used as initiator instead of the liquid MeOTf. The use of solid initiator enables more precise sampling, which is important for smaller batches to retain an accurate DP of copolymers.

Gradient copolymers often show a different behavior than random copolymers; they can cause for instance self-assembly in certain solvents [30], similarly to block copolymers. The gradient structure could also presumably influence the properties of further crosslinked gels, especially if the functional monomer is also highly hydrophobic. In case of more random distribution of functional crosslinking units within the chain, the intermolecular crosslinking is expected. On the other hand, if the polymer chains are crosslinked during self-assembly with the coumarin units forming the inner “block”, the units would crosslink inside the core of the self-assembled structure. That means, no typical gel consistency would be achieved as the micelles are not crosslinked with each other.

The aim was to decrease the probability of self-assembly formation to achieve more regular hydrogel networks. For the comparison, we prepared coumarin-modified copolymer by hydrolysis of poly(2-ethyl-2-oxazoline) (**PEtOx**, DP 100) and subsequent post-polymerization modification of the secondary amines with **2**, as described by Chujo et al. [6] (**PEtOx_modif**). The prepared copolymers were analyzed by ^1^H NMR spectroscopy and gel permeation chromatography (GPC). The analytical data of the prepared copolymers, the PEtOx homopolymer and the modified PEtOx, are summarized in Table 2. The experimental number average molar masses ($\overline{M }$_n_) from GPC measurements correspond relatively well to theoretical values. Also the dispersities are mostly below 1.5, as expected for living polymerization. The only exception was copolymer **PEtOx_Coum8** with slightly higher dispersity of 1.51, what can be caused due to the relatively high coumarin content. Further, for **PEtOx_Coum8** and **PEtOx_Coum2** a significantly higher coumarin content was expected according to the theoretical composition (8.00% instead of 6.37% and 1.85% instead of 1.27%). The coumarin content of **PEtOx_Coum4** is the nearest to the theoretical composition (3.20% instead of 3.50%), additionally to the lowest dispersity.

**Table 2:** Analytical data of block copolymers

Sample	Theoretical composition	$\overline{\mathrm{M} }$_n_ (theor)/kg mol^−1^	$\overline{\mathrm{M} }$_n_ (exp)/kg mol^−1^	Đ	F_coum_	Yield/%
PEtOx_Coum2	PEtOx_106_-*g*-PCoumOx_2_	11.0	6.8	1.18	1.27	85
PEtOx_Coum4	PEtOx_110_-*g*-PCoumOx_4_	11.9	10.5	1.07	3.20	80
PEtOx_Coum8	PEtOx_92_-*g*-PCoumOx_8_	11.2	9.8	1.51	6.37	97
PEtOx		10.2	12.8	1.36	–	85
PEtOx_modif					6.79	53

Theoretical composition and number average molar mass ($\overline{M }$_n_, theor) calculated from the feed; experimental number average molar mass ($\overline{M }$_n_, exp) and dispersity (Đ) measured by GPC, coumarin molar fraction (F_coum_) calculated from ^1^H NMR spectra

The original homopolymer **PEtOx** for the modified sample **PEtOx_modif** was hydrolyzed to reach 10% ethyleneimine units. 68% of the ethyleneimine units could be modified with coumarin derivatives. Therefore, the polymer chains exhibited 6.79% coumarin-containing units and 3.21% not modified ethyleneimine units. It is assumed that the DP as well as the experimental number average molar mass stayed unchanged after the modification.

### Thermoresponsive properties

**Fig. 3:**
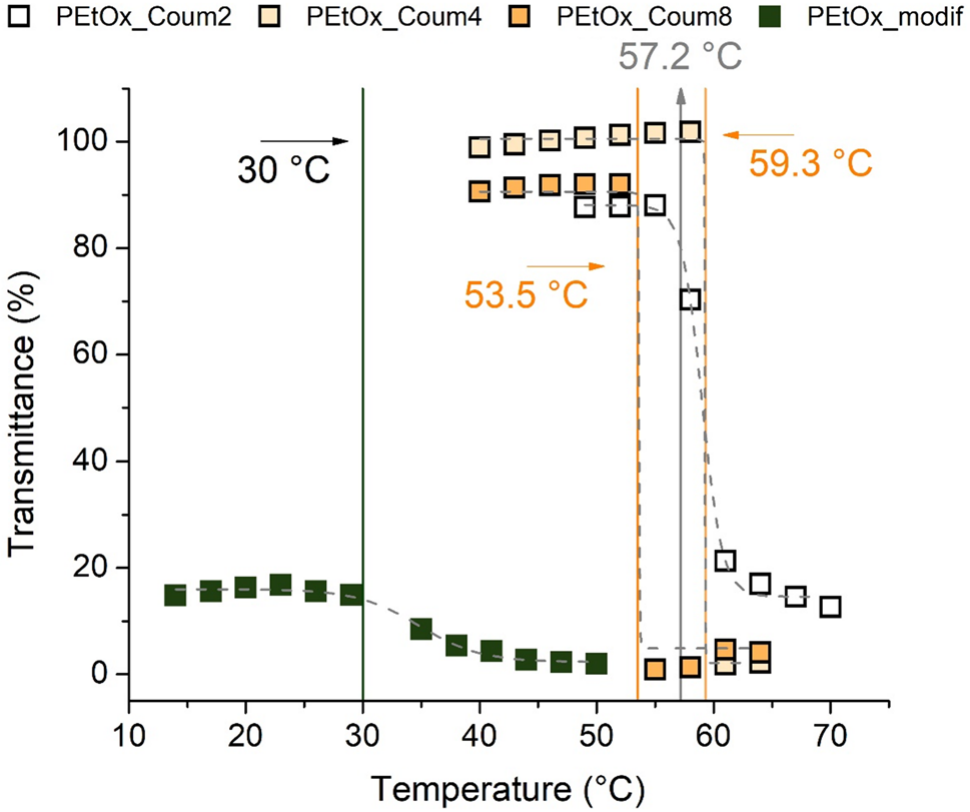
Dependence of transmittance on temperature for aqueous solutions (*c* = 10 mg cm^−3^) of gradient copolymers **PEtOx_Coum2**, **PEtOx_Coum4** and **PEtOx_Coum8** and modified copolymer **PEtOx_modif** measured by UV/Vis spectrometry. The grey dashed line represents Boltzmann fit, *T*_cp_ values were calculated as 90% transmittance from Boltzmann fit

As several poly(2-oxazoline) (POx) homopolymers and copolymers exhibit thermoresponsive behavior in aqueous solutions [31, 32], we decided to study this characteristic also for our newly synthesized gradient copolymers. The homopolymer PEtOx with a degree of polymerization (DP) more than 100 exhibits so-called *lower critical solution temperature* (LCST) behavior. Above certain critical temperature (cloud point temperature, *T*_cp_), the polymer becomes insoluble and precipitates from the solution, what reflects in decreased transmittance of the polymer solution. *T*_cp_ depends on the polymer concentration in solution, while the minimum value from phase diagram is referred to as LCST. Addition of hydrophobic comonomers into the polymer chain decrease the *T*_cp_ values, as shown by Hoogenboom et al. [33] and Tiller et al. [34], among others. The dependence of transmittance on temperature for aqueous solutions of **PEtOx_Coum2**, **PEtOx_Coum4**, **PEtOx_Coum8**

 and **PEtOx_modif** at the concentration 10 mg cm^−3^ is shown in Fig. 3. The newly synthesized gradient copolymers are all well-soluble in water at room temperature; however, they exhibit *T*_cp_ in the range from 53.5 °C to 59.3 °C. In comparison, PEtOx homopolymer with DP 100 exhibits *T*_cp_ around 90 °C at the concentration 10 mg cm^−3^ in water [33]. The presence of hydrophobic **CoumOx** thus causes the shift of *T*_cp_ to lower temperatures. While both **PEtOx_Coum4** and **PEtOx_Coum8** exhibit sharp transitions, **PEtOx_Coum2** exhibits broader transition with the *T*_cp_ value surprisingly lower than less hydrophilic **PEtOx_Coum4** with higher coumarin content. This effect can be caused by less defined copolymer composition, although the dispersity is relatively low (see Table 2). As a comparison, we also studied the thermoresponsive behavior of **PEtOx_modif**. However, the copolymer was already turbid at low temperatures, with maximum transmittance of around 20%. Also for this sample, we observed further decrease of transmittance with heating, which allows us to fit the measured points with Bolzmann fit. The obtained value of *T*_cp_ was 30 °C, much lower than the series of gradient copolymers. It should be noted that the measured values of *T*_cp_ of the newly synthesized gradient copolymers are above the human body temperature, relevant for biomedical application. However, as we have shown in our previous work, encapsulation of hydrophobic drug into amphiphilic POx leads to the decrease of *T*_cp_ [35]. In that case, especially copolymers with higher *T*_cp_ would be indeed interesting for further use, although their thermoresponsive behavior should be studied separately for targeted drug.

### Hydrogel preparation and swelling studies

Inspired by the pioneering work of Chujo et al. [6], we decided to exploit our copolymers for the preparation of hydrogels by crosslinking with UV-light. Polymers with coumarin moieties can be crosslinked by irradiation with UV light above 360 nm as coumarin groups form dimers (see Scheme 2a) [7]. The mechanism is a [2 + 2] cycloaddition of the double bonds that can be additionally cleaved by irradiation with light below 280 nm. As the coumarin dimerization is reversible, but both reactions are photoorthogonal, side reactions such as photocleavage could be excluded by using wavelengths above 320 nm.

**Figure:**
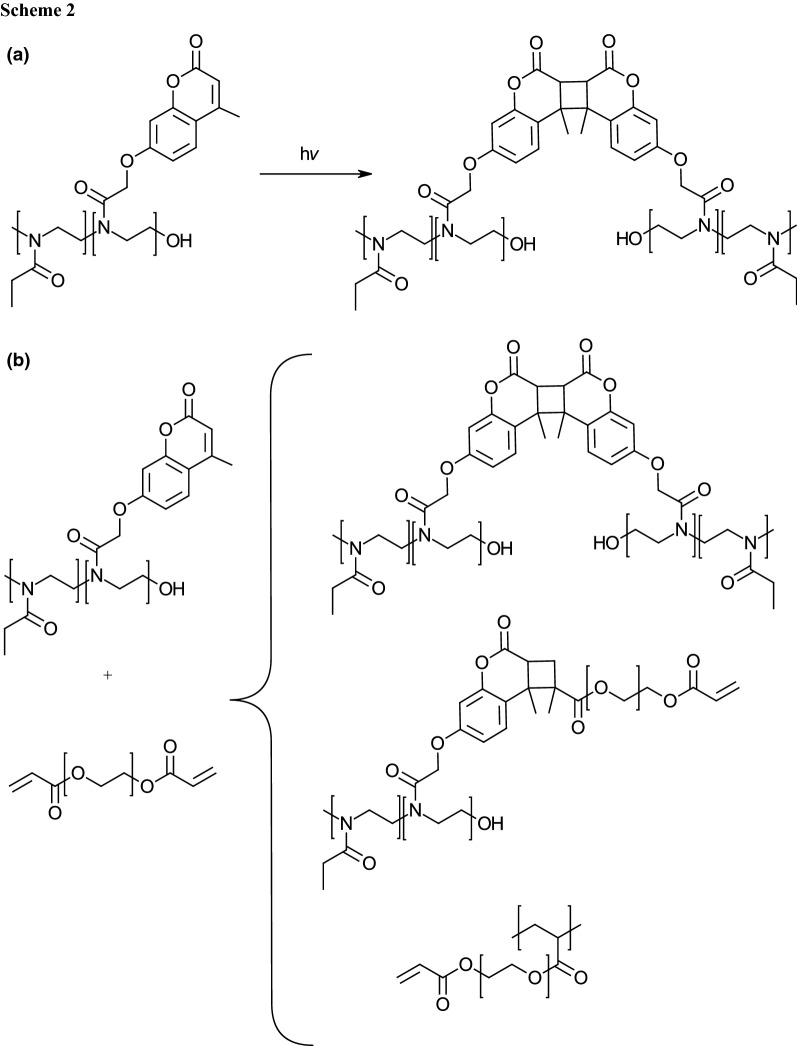

First, we started with some photorheology measurements using copolymer formulations of **PEtOx_Coum2**, **PEtOx_Coum4** and **PEtOx_Coum8** in DMSO and **PEtOx_modif** in 1,4-dioxane (each 25 wt.%), without any photoinitiator. We selected DMSO as a (relatively) good solvent for both “blocks”, to prevent formation of self-assembled structures. Unfortunately, no gels could be obtained as the light intensity is limited due to the experimental set-up. For more details regarding the photorheology, see Supporting Information chapter 4. Therefore, the following gelation experiments were conducted using the light source directly to reach higher light intensities.

The irradiation time and intensity were based on literature [5, 6, 36] where the ideal combination for the samples with the higher coumarin content, **PEtOx_Coum8** and **PEtOx_modif**, showed to be 2.51 W cm^−2^ for 30 min. For lower intensity as for shorter irradiation time, only pieces of gel could be obtained instead of a whole round gel. For higher intensity (up to 5 W cm^−2^) or also for longer irradiation time (up to 1 h), the result was a black solid without any solvent left.

While the copolymers with highest coumarin content (i.e., **PEtOx_Coum8**, and **PEtOx_modif**) indeed formed insoluble networks, the copolymers with lower coumarin content did not crosslink even after changing the irradiation parameters to longer irradiation times or higher intensity.

To enable crosslinking of our copolymers with lower coumarin content, we got inspired by a recent work of Eng et al. [36]. The authors described a system containing both coumarin and methacrylate containing polymers that can be crosslinked by irradiation via three different mechanisms simultaneously, as shown similarly in Scheme 2b with acrylates. This reaction is much faster than the [2 + 2] cycloaddition of two coumarin moieties.

As shown in Scheme 2b, instead of the before mentioned methacrylates, acrylates were used in further experiments. The cause was the higher reactivity of acrylates compared to methacrylates, although the next step would be gel formation in combination with methacrylates to improve the biocompatibility as acrylates show higher cytotoxicity. Conducting additionally similar experiments with methacrylates was beyond the scope of this paper.

We thus introduced poly(ethylene glycol) diacrylate (PEGDA) into our formulations, in the ratio 12.5 wt.% PEGDA to 12.5 wt.% of POx copolymer in DMSO. This composition led to the fast crosslinking without photoinitiator in case of all POx copolymers and formation of stable hydrogels. Pure PEGDA sample was also prepared as a control.

**Fig. 4:**
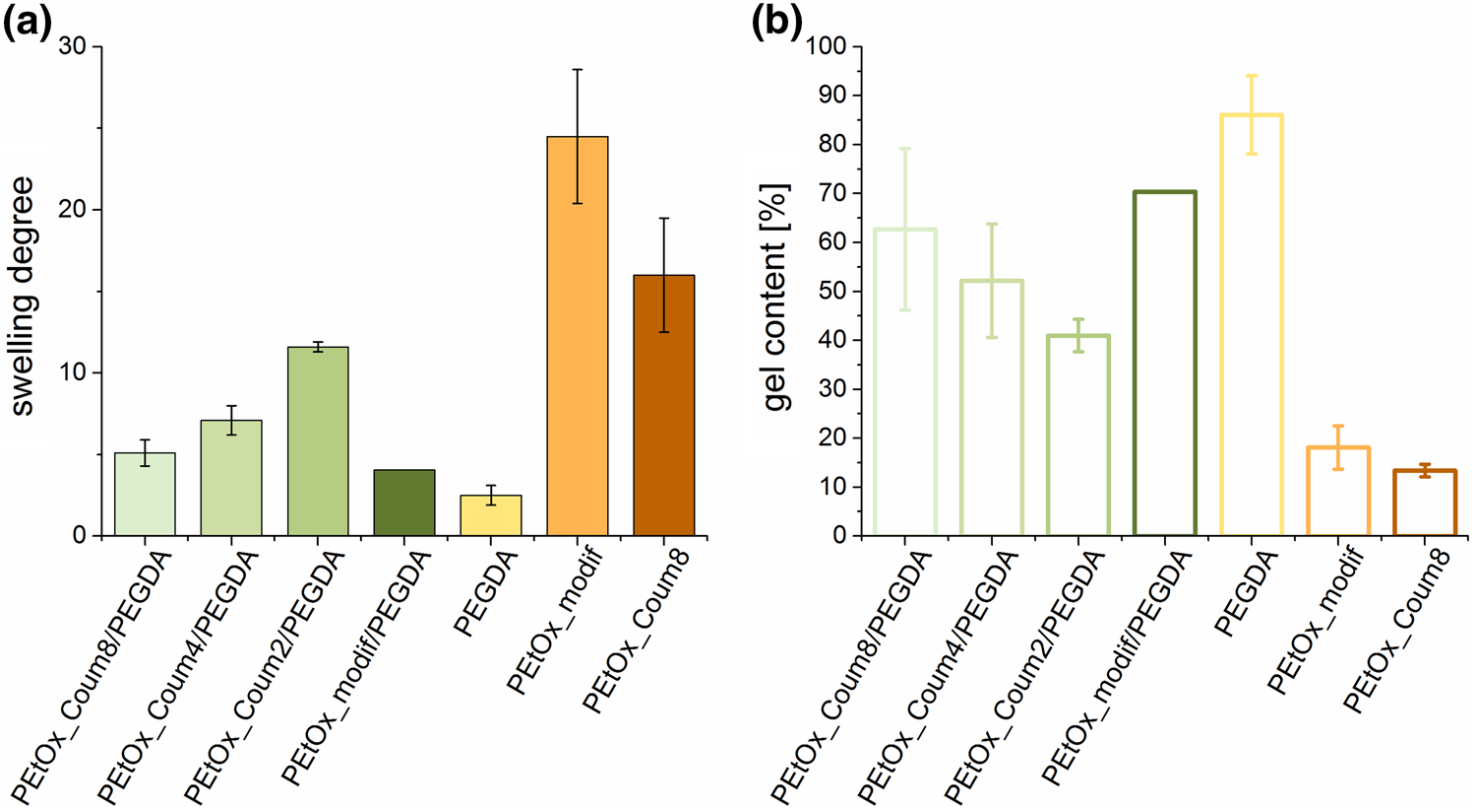
**a** Swelling degree and **b** gel content of all types of crosslinked hydrogels as follows: coumarin-containing polymers with PEGDA (green), pure PEGDA gels (yellow) and pure coumarin-containing polymer (orange) (color figure online)

Further, we studied the swelling properties in distilled water and the gel content for the series of prepared hydrogels. As shown in Fig. 4a, the swelling degree of the hydrogels containing **PEtOx_Coum2**, **PEtOx_Coum4** and **PEtOx_Coum8** in combination with PEGDA is increasing with decreasing coumarin content. This can be explained by the lower crosslinking density of POx with lower coumarin content, in combination of higher overall hydrophilicity of the POx copolymer and the decreasing gel content (see Fig. 4b). The gel containing **PEtOx_modif** in combination with PEGDA shows a similarly low swelling degree as the pure PEGDA hydrogel. Compared to the PEGDA containing gels, the ones consisting of pure POx show both a significantly higher swelling degree. Comparing here **PEtOx_modif** with the copolymerized sample **PEtOx_Coum8**, the trend is inversed to the PEGDA containing gels, as the modified sample achieved an even higher swelling degree than the copolymerized sample. Since their coumarin content is approximately the same, the difference is only due to the coumarin distribution along the polymer backbone. **PEtOx_modif** features more even distribution of coumarin groups along the polymer backbone, compared to the gradient copolymer **PEtOx_Coum8**. The gradient structure may lead to uneven crosslinking, with higher crosslinking densities on one side of the polymer chain and a flanking hydrophilic PEtOx “block” on the other side of the polymer chain.

As already mentioned, the gel content displayed in Fig. 4b is decreasing for the gels containing POx in combination with PEGDA with decreasing coumarin content. The change in the gel content was expected as less functional groups result in a lower crosslinking density. The highest gel content is achieved for the pure PEGDA gel, as the crosslinking mechanism is the very fast polymerization of the acrylate groups. The gels containing pure POx show a similar gel content, **PEtOx_modif** with a slightly higher value.

In general, the hydrogels with lower the gel content display a higher swelling degree. A higher crosslinking density results in a higher gel content, but also in a lower swelling degree, as a high crosslinking density decreases the water uptake.

## Conclusion

In summary, we synthesized a novel coumarin-containing 2-oxazoline in four steps starting with 4-methylumbelliferone. The homo-and copolymerization kinetics with EtOx of the novel monomer were investigated, where an inversion of the polymerization rates was observed. Compared to EtOx, the homopolymerization of **CoumOx** was faster. On the other hand, during the copolymerization, **CoumOx** exhibited lower *k*_p_ then EtOx, what was assumed to be the explanation for the experienced gradient polymer behavior. Further, three gradient copolymers containing **CoumOx** and EtOx were synthesized with different composition (2, 4 and 8 mol% coumarin content). For comparison, a sample was synthesized via post-polymerization modification according to Chujo et al. [6] with 8 mol% coumarin content.

The thermoresponsive behavior of the copolymers compared to the modified sample was studied in water. The copolymerized samples showed *T*_cp_ above body temperature. Compared to **PEtOx_modif**, all of the copolymerized samples had a sharper transition and a significantly higher *T*_cp_ (53.5–59.3 °C compared to 30 °C).

The polymers were crosslinked from a solution of 25 wt.% polymer in DMSO via irradiation to achieve hydrogels. Therefore, the two different POx copolymers (**PEtOx_modif** and **PEtOx_Coum8**, both with 8 mol% coumarin) were crosslinked without additives. **PEtOx_modif**, **PEtOx_Coum2**, **PEtOx_Coum4** and **PEtOx_Coum8** were also crosslinked by adding PEGDA (1:1 wt. ratio). The swelling degree of all gels were investigated, resulting in an increasing swelling degree with decreasing coumarin content for PEGDA-containing samples due to a decreasing crosslinking density. Both of the pure POx gels without PEGDA were found to show significantly higher swelling degrees, where **PEtOx_modif** exhibited the highest swelling degree due to its even distribution of the coumarin groups.

Here, we prepared new gradient POx copolymers with different coumarin content. Since the synthesized copolymers exhibit thermoresponsive behavior in water, which can be tuned by the copolymer composition, and form gels upon irradiation, they could find a potential use in biomedical application. However, the cytotoxicity tests have to be conducted in the future to ensure the biocompatibility of prepared materials.

## Experimental

2-Ethyl-2-oxazoline (Sigma-Aldrich), 2-methyl-2-oxazoline (Sigma–Aldrich) and acetonitrile (Acros Organics) were dried over CaH_2_ overnight, distilled under argon and stored over molecular sieves (3 Å, Merck). Methyl triflate (MeOTf, purchased from Sigma-Aldrich) was distilled via Kugelrohr distillation and stored under argon. The low water content of all starting compounds was ensured by Karl Fischer titrations using an Envirotech CA-21 moisture meter (Aquamicron™).

4-Methylumbelliferone was purchased from Sigma–Aldrich and used as received. Ethyl bromoacetate was purchased from Fluka and distilled in vacuo prior to use. Acetone was dried with anhydrous K_2_CO_3_ overnight, distilled under argon and stored over molecular sieve. Dioxane was purchased from Acros Organics and used as received. Triethylamine (TEA) was purchased from Roth, distilled under argon and stored over molecular sieves protected from light. Isobutyl chloroformate and chloroethylamine hydrochloride were purchased from Sigma-Aldrich and stored under argon. Dry DMF was purchased from Acros Organics and stored over molecular sieves under argon. K_2_CO_3_ and *p*-anisic acid were purchased from Merck and used as received.

Commercial grade dichloromethane (DCM, Donau Chemie), methanol (MeOH, Donau Chemie) and dioxane (Donau Chemie) were dried using a PureSolv system (Inert, Amesbury, MA). Chloroform-*d*, DMSO-*d*_*6*_ and methanol-*d*_*4*_ were purchased from Eurisotop and used as received.

### Monomer and (co)polymer characterization methods

HR-MS analysis was performed from the monomer samples, dissolved in HPLC-grated acetonitrile (concentration: 10 μM), by using an HTC PAL system autosampler (CTC Analytics AG, Zwingen, Switzerland), an Agilent 1100/1200 HPLC with binary pumps, degasser, and column thermostat (Agilent Technologies, Waldbronn, Germany) and an Agilent 6230 AJS ESI-TOF mass spectrometer (Agilent Technologies, Palo Alto, CA).

NMR spectra were recorded on a Bruker DPX-200 FT NMR spectrometer at 200 MHz for ^1^H and 50 MHz for ^13^C, as well as on a Bruker Avance DRX-400 FT NMR spectrometer at 400 MHz for ^1^H and 100 MHz for ^13^C. The signals are recorded according to their chemical shifts, which were reported in ppm (s = singlet, d = doublet, t = triplet, q = quartet, qn = quintet, sep = septet, m = multiplet, bs = broad singlet) in comparison to tetramethyl silane (*δ* = 0 ppm). The spectra were then referenced on the used NMR-solvent [^1^H: CDCl_3_ (7.26 ppm), DMSO-*d*_*6*_ (2.50 ppm), MeOD (3.3 ppm), ^13^C: CDCl_3_ (77.16 ppm)]. Analysis of the spectra was performed with the program MestRe Nova v12.0.4 from Mestrelab Research S.L.

Melting Points (or decomposition onsets) were conducted with an OptiMelt Automated Melting Point System (Standford Research System) at a heating rate of 1 °C min^−1^.

Gel permeation chromatography (GPC) measurements were carried out in dry THF (stabilized with 250 ppm butylated hydroxytoluene (BHT) to hinder the formation of radicals) Malvern VISCOTEK TDA system containing a ViscotekTDA 305–021 RI + Viscodetector, a UV Detector Module 2550 for TDA 305 and a VISCOTEK SEC-MALS 9 light scattering detector was used. Separation was done with three series-connected PSS SDC columns with particle sizes 100 Å, 1000 Å and 100,000 Å with a flow rate of 0.8 cm^3^ min^−1^ at isothermal conditions at 35 °C. Conventional calibration is done following a calibration curve from the measurements of 11 narrow polystyrene standards produced by PSS. Samples were prepared as 1–3 mg cm^−3^ solutions in hexafluorisopropanol (HFIP):THF 1:9 mixture that were syringe-filtered. For analysis of the elugrams the software OmniSEC V5.12.461 from Malvern was used.

#### Ethyl (4-methylcoumarin-7-yloxy)acetate (1)

12.78 g 4-Methylumbelliferone (72.5 mmol, 1 eq.) was dried in a three-necked flask and flushed with argon. It was dissolved in 300 cm^3^ dry acetone, then 8.9 cm^3^ freshly distilled ethyl bromoacetate (80.5 mmol, 1.1 eq.) and 10.55 g anhydrous K_2_CO_3_ (76.3 mmol, 1.05 eq.) were added under stirring according to Chujo et al. [6]. The mixture was refluxed for three hours, resulting in a turbid yellow suspension. The precipitate was filtered and the solvent of the filtrate was evaporated in vacuo. The viscous yellow liquid was cooled to 0 °C to crystallize the crude product, afterwards it was recrystallized from ethanol to yield 13.10 g (69%) of white crystalline needles. The synthesis was performed in a yellow light laboratory. All windows of this laboratory were covered with adhesive foils (company: IFOHA), and the fluorescent lamps were type Osram lumilux with chip control light colour 62 (filtration of wavelengths below 480 nm). M.p.: 103.1–104.5 °C; ^1^H NMR (200 MHz, CDCl_3_): *δ* = 7.52 (d, *J* = 8.8 Hz, 1H, Ar), 6.90 (t, *J* = 2.7 Hz, 1H, Ar), 6.78 (d, *J* = 2.6 Hz, 1H, Ar), 6.16 (q, *J* = 1.2 Hz, 1H, Ar), 4.68 (s, 2H, CH_2_), 4.26 (dq, *J* = 10.2, 7.1 Hz, 2H, CH_2_), 2.40 (d, *J* = 1.3 Hz, 3H, CH_3_), 1.30 (td, *J* = 7.1, 2.6 Hz, 3H, CH_3_) ppm.

#### (4-Methylcoumarin-7-yloxy)acetic acid (2)

5.07 g Sodium hydroxide (137.1 mmol, 14.4 eq.) and 2.50 g **1** (9.5 mmol, 1 eq.) were dissolved in each 100 cm^3^ water and dioxane [6]. The solution was stirred overnight and the solvent was evaporated in vacuo, leaving a white-yellow solid. The residue was dissolved in 20 cm^3^ distilled water and acidified with concentrated HCl. The product precipitated as a beige voluminous solid that was filtrated, washed with 200 cm^3^ cold water and dried at 60 °C overnight. 2.14 g of a beige solid (96% yield) were obtained. M.p.: 195.0–210.0 °C; ^1^H NMR (400 MHz, DMSO-*d*_*6*_): *δ* = 12.97 (s, 1H, COO*H*), 7.69 (d, *J* = 8.5 Hz, 1H, Ar), 7.04–6.87 (m, 2H, Ar), 6.22 (d, *J* = 1.6 Hz, 1H, Ar), 4.83 (s, 2H, CH_2_), 2.40 (d, *J* = 1.4 Hz, 3H, CH_3_) ppm.

#### *N*-2-Chloroethyl-(4-methyl-7-coumarinyloxy)acetamide (3, C_14_H_14_ClNO_4_)

1 g **2** (4.27 mmol, 1 eq.) was dissolved in 45 cm^3^ dry dioxane and 0.56 cm^3^ TEA (0.48 mmol, 1.1 eq.) was added under argon atmosphere before the solution was cooled to 0 °C [26]. Isobutyl chloroformate (0.65 cm^3^, 4.27 mmol, 1 eq.) was added, receiving a turbid solution that was stirred 15 min at 0 °C and further 15 min at RT. After cooling it again to 0 °C, 0.61 g 2-chloroethylamine hydrochloride (5.12 mmol, 1.2 eq.) dissolved in 4 cm^3^ dry DMF and 0.56 cm^3^ TEA (0.48 mmol, 1.1 eq.) were added slowly. The suspension was stirred for 2 h at RT; afterwards the solvent was evaporated. The slightly yellow solid was dissolved in DCM and filtered. 100 cm^3^ diethyl ether were added and the white precipitate was filtered off. The product was precipitated by adding 100 cm^3^ of petrol ether, and the white solid was dried at 60 °C overnight (yield 6.45 g, 74%). M.p.: 158.4–208.8 °C (decomposition); ^1^H NMR (400 MHz, CDCl_3_): *δ* = 7.56 (d, *J* = 8.8 Hz, 1H, Ar), 6.91 (dd, *J* = 8.7, 2.6 Hz, 2H, Ar), 6.87 (d, *J* = 2.5 Hz, 1H, –NH), 6.19 (q, *J* = 1.3 Hz, 1H, –CO–CH =), 4.58 (s, 2H, –O–CH_2_–CO–), 3.77–3.64 (m, 4H, –CH_2_–CH_2_–Cl), 2.41 (d, *J* = 1.2 Hz, 3H, –CH_3_) ppm; ^13^C NMR (101 MHz, CDCl_3_): *δ* = 167.51 (quart. C), 160.95 (quart. C), 159.85 (quart. C), 155.26 (quart. C), 152.30 (quart. C), 126.19 (CH), 115.07 (quart. C), 113.11 (CH), 111.95 (CH), 102.69 (CH), 67.57 (CH_2_), 43.69 (CH_2_), 40.94 (CH_2_), 18.83 (CH_3_) ppm; HR-MS (ACN, ESI +): *m/z* calc. 296.7239 ([M + H]^+^), found 296.0686 (diff 0.38 ppm).

#### 2-[(4-Methyl-7-coumarinyloxy)methyl]oxazoline (4, CoumOx, C_14_H_13_O_4_N) 6.45 g 3

(21.8 mmol, 1 eq.) and 6.47 g anhydrous K_2_CO_3_ (45.8 mmol, 2.1 eq.) were flushed with argon before 87 cm^3^ dry MeOH was added [26]. The mixture was refluxed for 5 h and after cooling down to RT the solvent was evaporated. The residue was dissolved in DCM, centrifuged and the supernatant liquid was dried in vacuo to receive an off-white solid (yield 2.53 g, 45%). M.p.: 170.0–220.0 °C (decomposition); ^1^H NMR (400 MHz, CDCl_3_): *δ* = 7.52 (d, *J* = 8.8 Hz, 1H, Ar), 6.96 (dd, *J* = 8.8, 2.6 Hz, 1H, Ar), 6.89 (d, *J* = 2.5 Hz, 1H, Ar), 6.16 (q, *J* = 1.2 Hz, 1H, –CO–CH =), 4.78 (t, *J* = 1.4 Hz, 2H, –O–CH_2_–), 4.43–4.30 (m, 2H, –O–CH_2_–CH_2_–N), 4.02–3.89 (m, 2H, –O–CH_2_–CH_2_–N), 2.40 (d, *J* = 1.3 Hz, 3H, –CH_3_) ppm; ^13^C NMR (101 MHz, CDCl_3_): *δ* = 163.08 (quart. C), 161.21 (quart. C), 160.94 (quart. C), 155.19 (quart. C), 152.52 (quart. C), 125.85 (CH), 114.48 (quart. C), 112.70 (CH), 112.58 (CH), 102.01 (CH), 68.29 (CH_2_), 62.88 (CH_2_), 54.64 (CH_2_), 18.79 (CH_3_) ppm; HR-MS (ACN, ESI +): *m/z* calc. 282.2458 ([M + Na]^+^), found 282.2798 (diff 2.35 ppm).

### Homopolymerization

**CoumOx** (0.340 g, 1.31 mmol, 50 eq.) was weighted into a vial, flushed with argon and dried carefully in HV for 30 min. 0.43 cm^3^ of initiator stock solution (10 mg cm^−3^ of MeOTf in dry acetonitrile; 0.026 mmol, 1 eq.) was added. The suspension was put into a preheated oil bath at 75 °C, where the monomer dissolved. Samples were taken after 1, 2, 3, 5 and 7 h in the size of approximately 0.05 cm^3^ and analyzed via ^1^H NMR spectroscopy (integral monomer 4.27–4.20 ppm and 3.86–3.76 ppm, integral polymer 3.65–3.40 ppm). The general conditions of the polymerization were a 3-M solution of monomer in the respective solvent, the molar ratio of monomer to initiator was calculated to be 50.

After 28 h the polymerization was cooled to RT and terminated with 1.2 Eq. 1 M KOH in MeOH overnight. The solvent was evaporated to yield 312 mg (92%) an off-white solid, further purification and analysis were not possible due to the insolubility of the product. ^1^H NMR (400 MHz, CDCl_3_): *δ* = 7.41–7.28 (m, Ar), 6.92–6.47 (m, Ar), 6.01–5.75 (m, –CO–CH =), 5.01–4.54 (m, polymer backbone), 3.71–3.40 (m, –O–CH_2_–), 2.35–2.10 (m, –CH_3_) ppm.

### Copolymerization

#### Kinetic study

**CoumOx** (150 mg, 0.58 mmol, 10 eq.) was weighted into a Schlank flask and dried for 2 h on high vacuum. 2-Ethyl-2-oxazoline (229 mg, 2.31 mmol, 10 eq.) and dry acetonitrile was added until **CoumOx** was dissolved at 75 °C (2 cm^3^). MeOTf (9 mg, 0.06 mmol, 1 eq.) was added to start the polymerization. Samples for NMR analysis of 0.05 cm^3^ were taken over 4 days. The reaction was stopped after 11 days with 0.2 cm^3^ of 1 M KOH in MeOH and stirred for 24 h.

#### PEtOx_Coum2

The initiator MeONs (11.5 mg, 0.05 mmol, 1 eq) and 26 mg **CoumOx** (0.1 mmol, 2 eq) were weighted into the Schlenk flask. The reaction flask was subsequently dried for 2 h on high vacuum, then 1.5 cm^3^ acetonitrile and 0.53 g 2-ethyl-2-oxazoline (5.3 mmol, 106 eq) were added. The reaction mixture was polymerized for 10 days at 75 °C in an oil bath. After the full conversion was confirmed via NMR, the polymerization mixture was terminated by adding 0.1 cm^3^ of 1 M KOH in MeOH and stirred at RT for 24 h. Then, the polymers were precipitated in 100 cm^3^ of cold diethyl ether, dried under vacuum, re-dissolved in distilled water and dialysed (SpectraPor^®^ membrane of 1 kDa molecular weight cut-off, Spectrum Laboratories, Inc, USA) against distilled water overnight. The copolymer was obtained as off-white powder after freeze-drying using Christ freeze-drying system Gamma 2–20 at a pressure of ~ 0.5 mbar and a collector temperature of − 45 °C (yield 0.47 g, 85%).

#### PEtOx_Coum4

The initiator MeONs (11.5 mg, 0.05 mmol, 1 eq) and 55 mg **CoumOx** (0.21 mmol, 4.2 eq) were weighted into the Schlenk flask. The reaction flask was subsequently dried for 2 h on high vacuum, then 1.5 cm^3^ acetonitrile and 0.55 g 2-ethyl-2-oxazoline (5.5 mmol, 110 eq) were added. The reaction mixture was polymerized for 7 days at 75 °C in an oil bath. After the full conversion was confirmed via NMR, the polymerization mixture was terminated by adding 0.1 cm^3^ of 1 M KOH in MeOH and stirred at RT for 5 h. Then, the polymers were precipitated in 100 cm^3^ of cold diethyl ether, dried under vacuum, re-dissolved in distilled water and dialysed (SpectraPor^®^ membrane of 1 kDa molecular weight cut-off, Spectrum Laboratories, Inc, USA) against distilled water overnight. The copolymer was obtained as off-white powder after freeze-drying (yield 0.486 g, 80%).

#### PEtOx_Coum8:

**CoumOx** (387 mg, 1.49 mmol, 8 eq) was weighted into the Schlenk flask and was dried for 2 h on high vacuum. Acetonitrile (6.2 cm^3^), 1.718 g 2-ethyl-2-oxazoline (17.15 mmol, 92 eq) and 30.66 mg MeOTf (0.19 mmol, 1 eq.) were added. The reaction mixture was polymerized for 7 days at 75 °C in an oil bath. After the full conversion was confirmed via NMR, the polymerization mixture was terminated by adding 0.5 cm^3^ of 1 M KOH in MeOH and stirred at RT overnight. Then, the polymers were precipitated in 100 cm^3^ of cold diethyl ether, dried under vacuum at 50 °C, re-dissolved in MeOH:CHCl_3_ 2:1 and precipitated again. After drying in vacuum at 50 °C, the copolymer was obtained as orange solid (yield 2.08 g, 97%).

### Hydrolysis and post-polymerization modification

#### Partial hydrolysis of PEtOx

PEtOx (2.70 g, 27.2 mmol) was dissolved in 57 cm^3^ water (0.48 M amide concentration) and 5.2 cm^3^ concentrated hydrochloric acid was added to receive a 1 M acidic solution [37]. The flask was placed in a preheated oil bath (110 °C) immediately to start the hydrolysis. After carefully selected time intervals to reach a selected degree of hydrolysis (DH), the solution was cooled rapidly to RT with an ice bath. 1 M aqueous sodium hydroxide was added to the polymer solution until a pH of 8–10 was reached. The product was purified by dialysis for 24 h, frozen with liquid nitrogen and lyophilized (yield 2.66 g, 99%). ^1^H NMR (400 MHz, CD_3_OD): *δ* = 3.8–3.4 (m, 4H, PEtOx backbone), 3.2–2.65 (m, 4H, PEI backbone), 2.55–2.3 (m, 2H, CO–CH_2_–CH_3_ side chain), 1.2–1.0 (m, 3H, –CH_3_) ppm.

#### Modification of partially hydrolyzed PEtOx

The partially hydrolyzed PEtOx (1.97 g, DH 10%, 1 eq. amine) and 1.00 g **2** (4.3 mmol, 2 eq.) were weighted into a three-necked flask and dried over HV. The solids were dissolved in 64 cm^3^ dry DCM and 19 cm^3^ dry DMF and cooled to 0 °C. After 1.34 g DCC (6.5 mmol, 3 eq.) was added rapidly, the solution was stirred overnight at room temperature. The off-white precipitate was filtered, washed with DCM and the solvent of the yellow filtrate was evaporated in vacuo, resulting in a pale orange residue. After dissolving the solid in 100 cm^3^ methanol, it was precipitated into 300 cm^3^ diethyl ether to receive an orange solid (yield 1.12 g, 38%).

### Thermoresponsive properties

The thermoresponsive properties of aqueous solutions gradient copolymers **PEtOx_Coum2**, **PEtOx_Coum4**, **PEtOx_Coum8** and a modified copolymer **PEtOx_modif** were studied by measuring transmittance as a function of temperature by UV/Vis spectrometer. A UV-1800 instrument (Shimadzu, Kyoto, Japan) equipped with a six-cell thermoelectrical temperature controller CPS-240A (Shimadzu, Kyoto Japan) was used for measuring the UV/Vis spectra. The samples were dissolved in distilled water at the concentration 10 mg cm^−3^ prior to measurement. The measurement was performed in the temperature range of 14–70 °C with a 3 °C step. The equilibration time was 5 min. The transmittance at a wavelength of 500 nm was evaluated. The cloud point temperature (*T*_cp_) was calculated from Bolzmann fit of the transmittance curves at 90% decrease of original transmittance values.

### Hydrogel preparation

For the preparation of hydrogels, a stock solution of 25 wt.% of each the copolymerized coumarin-containing copolymers (**PEtOx_Coum2**, **PEtOx_Coum4** and **PEtOx_Coum8**) in DMSO and **PEtOx_modif** in 1,4-dioxane was prepared. In addition, 25 wt.% in DMSO of PEGDA 700 and mixtures of PEGDA 700:POx (with each **PEtOx_modif** and **PEtOx_Coum8**) in ratios 12.5 wt.%:12.5 wt.% were prepared. 50 mm^3^ of copolymer stock solution was pipetted into circular silicon mold (diameter 10 mm) and closed with a cover glass. The solution was then irradiated for 30 min with UV light from the distance 1.8 cm (OmniCure 2000, equipped with a 320–500 nm filter and a single-tube liquid filled light guide with a diameter of 8 mm, intensity 2.51 W cm^−2^ at the tip of the lightguide, calibrated with an Omnicure R2000 radiometer).

### Swelling studies

After the irradiation, the material was transferred into vial filled with distilled water and allowed to swell for 24/48 h. After this time interval, the crosslinked hydrogels were withdrawn from the solution, gently dried with tissue paper and weighted. Subsequently, they were frozen, freeze-dried and their weight was measured again. The swelling degree (SD) was calculated as follows according to Eq. (1):

1 $$SD = \frac{{m_{sw} - m_{d} }}{{m_{d} }},$$

where *m*_sw_ is the weight of swollen gel and *m*_d_ is the weight of dried gel. The gel content (GC) was calculated using Eq. (2) as follows:

2 $${\text{GC}}\;\left( \% \right) = \frac{{{\text{m}}_{{\text{d}}} \cdot 100}}{{{\text{m}}_{{\text{f}}} \cdot {\text{c}}}}$$

with *m*_f_ as the weight of the used formulation and *c* as the polymer concentration in the formulation in %.

## Supplementary Information

Below is the link to the electronic supplementary material.

Supplementary file1 (PDF 1822 KB)

## Data Availability

The data that support the findings of this study are available from the corresponding author, S.B., upon reasonable request.

## References

[1] Dargaville TR, Park J-R, Hoogenboom R (2018). Macromol Biosci.

[2] Kelly AM, Wiesbrock F (2012). Macromol Rapid Commun.

[3] Highley CB, Rodell CB, Burdick JA (2015). Adv Mater.

[4] https://gestis.dguv.de/data?name=490110, 14.07.2022

[5] Nagata M, Yamamoto Y (2008). React Funct Polym.

[6] Chujo Y, Sada K, Saegusa T (1990). Macromolecules.

[7] Kabb CP, O’Bryan CS, Deng CC, Angelini TE, Sumerlin BS (2018). ACS Appl Mater Interfaces.

[8] Zahoranová A, Kroneková Z, Zahoran M, Chorvát D, Janigová I, Kronek J (2016). J Polym Sci. Part A:.

[9] Viegas TX, Bentley MD, Harris JM, Fang Z, Yoon K, Dizman B, Weimer R, Mero A, Pasut G, Veronese FM (2011). Bioconjugate Chem.

[10] Luxenhofer R, Schulz A, Roques C, Li S, Bronich TK, Batrakova EV, Jordan R, Kabanov AV (2010). Biomaterials.

[11] Leiske MN, Lai M, Amarasena T, Davis TP, Thurecht KJ, Kent SJ, Kempe K (2021). Biomaterials.

[12] Pizzi D, Mahmoud AM, Klein T, Morrow JP, Humphries J, Houston ZH, Fletcher NL, Bell CA, Thurecht KJ, Kempe K (2021). Eur Polym J.

[13] Finnegan JR, Pilkington EH, Alt K, Rahim MA, Kent SJ, Davis TP, Kempe K (2021). Chem Sci.

[14] Hsiue G-H, Chiang H-Z, Wang C-H, Juang T-M (2006). Bioconjugate Chem.

[15] Mero A, Pasut G, Via LD, Fijten MWM, Schubert US, Hoogenboom R, Veronese FM (2008). J Controlled Release.

[16] Moreadith RW, Viegas TX, Bentley MD, Harris JM, Fang Z, Yoon K, Dizman B, Weimer R, Rae BP, Li X, Rader C, Standaert D, Olanow W (2017). Eur Polym J.

[17] https://clinicaltrials.gov/ct2/show/NCT02579473, 08.12.2021

[18] Seeliger W, Aufderhaar E, Diepers W, Feinauer R, Nehring R, Thier W, Hellmann H (1966). Angew Chem Int Ed.

[19] Kagiya T, Narisawa S, Maeda T, Fukui K (1966). J Polym Sci Part B: Polym Lett.

[20] Tomalia DA, Sheetz DP (1966). J Polym Sci, Part A-1.

[21] Bassiri TG, Levy A, Litt M (1967). J Polym Sci Part B.

[22] Nahm D (2021) PhD Thesis, Julius-Maximilians-Universität Würzburg (Würzburg)

[23] Korchia L, Bouilhac C, Lapinte V, Travelet C, Borsali R, Robin J-J (2015). Polym Chem.

[24] Breunig M, Lungwitz U, Liebl R, Fontanari C, Klar J, Kurtz A, Blunk T, Goepferich A (2005). J Gene Med.

[25] Aoi K, Okada M (1996). Prog Polym Sci.

[26] Cesana S, Kurek A, Baur MA, Auernheimer J, Nuyken O (2007). Macromol Rapid Commun.

[27] Verbraeken B, Monnery BD, Lava K, Hoogenboom R (2017). Eur Polym J.

[28] Bouten PJM, Hertsen D, Vergaelen M, Monnery BD, Catak S, van Hest JCM, Van Speybroeck V, Hoogenboom R (2015). J Polym Sci. Part A.

[29] Hoogenboom R, Fijten MWM, Schubert US (2004). J Polym Sci. Part A.

[30] Datta S, Jutková A, Šrámková P, Lenkavská L, Huntošová V, Chorvát D, Miškovský P, Jancura D, Kronek J (2018). Biomacromol.

[31] Weber C, Hoogenboom R, Schubert US (2012). Prog Polym Sci.

[32] Oleszko-Torbus N, Utrata-Wesołek A, Wałach W, Dworak A (2017). Eur Polym J.

[33] Hoogenboom R, Thijs HML, Jochems MJHC, van Lankvelt BM, Fijten MWM, Schubert US (2008). Chem Commun.

[34] Hijazi M, Schmidt M, Xia H, Storkmann J, Plothe R, Santos DD, Bednarzick U, Krumm C, Tiller JC (2019). Polymer.

[35] Majerčíková M, Nádaždy P, Chorvát D, Satrapinskyy L, Valentová H, Kroneková Z, Šiffalovič P, Kronek J, Zahoranová A (2021). Polymers.

[36] Eng YJ, Xu J, Sugiarto S, Jonnalagadda US, Ang W, Lee JH-C, Kwan JJ, Nguyen TM (2021). Appl Polym Mater.

[37] de la Rosa VR, Bauwens E, Monnery BD, De Geest BG, Hoogenboom R (2014). Polym Chem.

