# Supplementary material for: Synthesis of coumarin-containing poly(2-oxazoline)s and light-induced crosslinking for hydrogel formation
Source: Monatsh Chem. 2022 Dec 8;154(5):459–71. doi: 10.1007/s00706-022-03013-8 (PMC10113345; doi:10.1007/s00706-022-03013-8)
Supplement: Supplementary file 1 — Supplementary file1 (PDF 1822 KB) [file 706_2022_3013_MOESM1_ESM.pdf]

Synthesis of Coumarin-containing Poly(2-oxazoline)s and Light-induced Crosslinking for Hydrogel Formation

Supporting Information

Carola Haslinger[1,2] ● Anna Zahoranová[2] ● Stefan Baudis[1,2]

[1] Christian Doppler Laboratory for Advanced Polymers for Biomaterials and 3D Printing, Getreidemarkt 9, 1060 Vienna, Austria

[2] Institute of Applied Synthetic Chemistry, Technische Universität Wien, Getreidemarkt 9, 1060 Vienna, Austria

🖂 Stefan Baudis, Stefan.baudis@tuwien.ac.at, www.baudislab.com

Table of Contents

1 ^{13}C NMR of Synthesized Monomer 2
2 Homopolymerization 3
3 Copolymerization 4
4 Crosslinking 7
5 Hydrogel formation and swelling studies 9
6 Literature 10

1 ^{13}C NMR of Synthesized Monomer

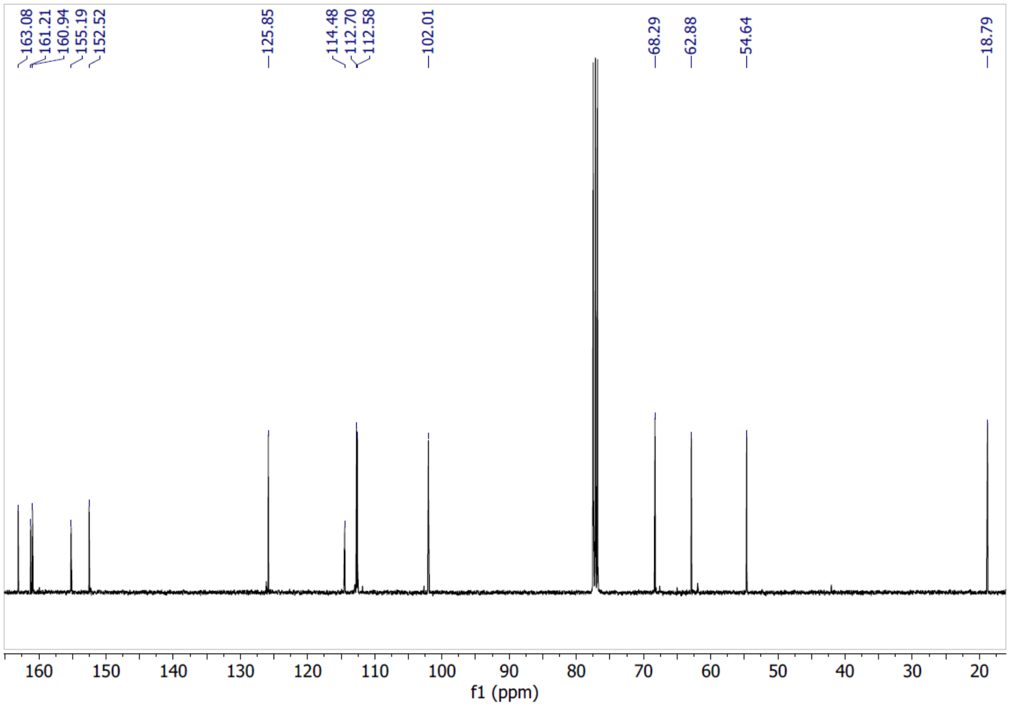

*Figure ESI 1. ^{13}C NMR spectrum of **CoumOx** in $CDCl_3$*

2 Homopolymerization

The samples drawn from reaction mixture were diluted and possible precipitates were immediately dissolved in $CDCl_3$ for measurement of ^1H NMR spectra. The polymer backbone (CH_2-CH_2) was found as a multiplet from approx. 3.65 – 3.40 ppm, the CH_2 signals from the monomer were both found as triplet from 4.27 – 4.21 ppm and 3.85 – 3.75 ppm. Equation 1 shows the calculation of the conversion using the mentioned signals in the ^1H NMR spectra.

$$Conversion\ (\%) = \frac{polymer\ backbone * 100}{(\sum monomer\ CH_2\ signals + polymer\ backbone)} \quad (1)$$

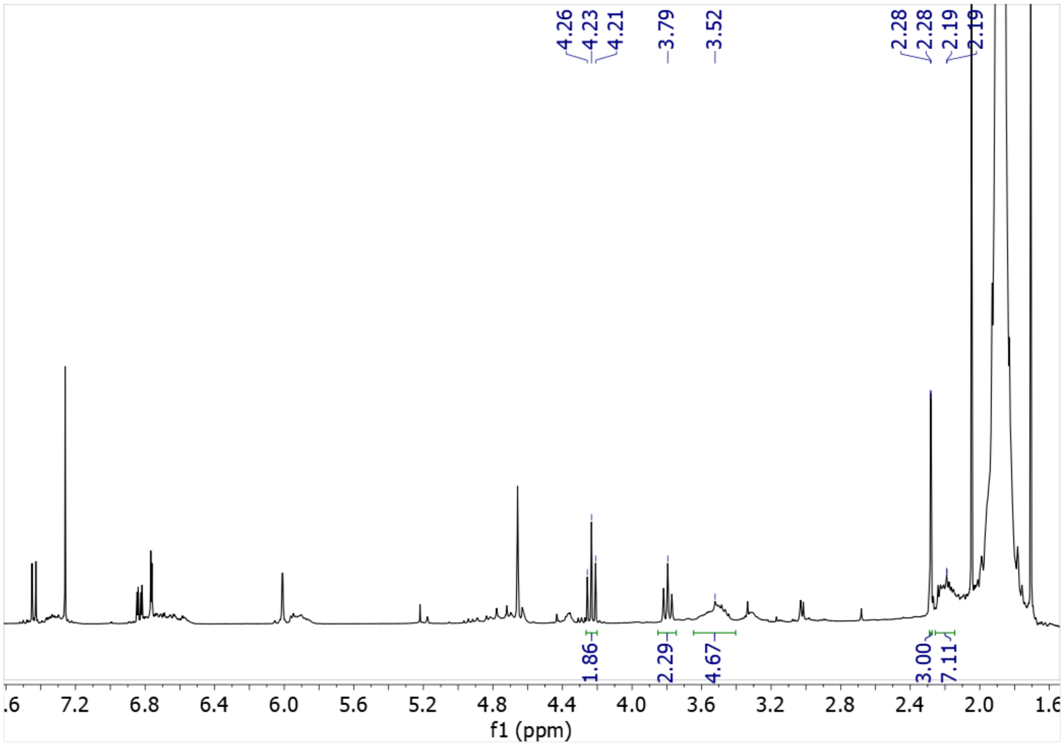

*Figure ESI 2. ^1H NMR spectrum in $CDCl_3$ for determining the homopolymerization kinetics of **CoumOx** after 2 h*

The homopolymerization kinetics of EtOx and MeOx were calculated using the same formula with the analogous polymer and monomer peaks from the ^1H NMR spectra.

3 Copolymerization

The coumarin content was calculated by normalizing the aromatic coumarin peak at approx. 7.50 ppm as 1H and using the polymeric backbone (singlet at approx. 3.4 ppm, 4H; equation 2) and the CH_3-ethyl group (at approx. 3.3 ppm, 3H; equation 3). Each polymer was known to contain of 100 monomer units. The results are displayed in Table ESI 1.

$$Coumarin\ content\ (mol\%) = \frac{100}{polymeric\ backbone/4} \tag{2}$$

$$Coumarin\ content\ (mol\%) = \frac{100}{ethyl\ group/3} \tag{3}$$

Table ESI 1. Calculation of the coumarin content of the copolymerized and the modified polymers from ^1H NMR spectra

	**Integrals**		**Coumarin content**	
	**backbone**	**CH_3 EtOx**	**(1) mol%**	**(2) mol%**
**PEtOx_Coum2**	465.63	236.85	0.86	1.27
**PEtOx_Coum4**	180.41	93.69	2.22	3.20
**PEtOx_Coum8**	85.81	47.08	4.66	6.37
**PEtOx_Modif**	79.59	44.21	5.03	6.79

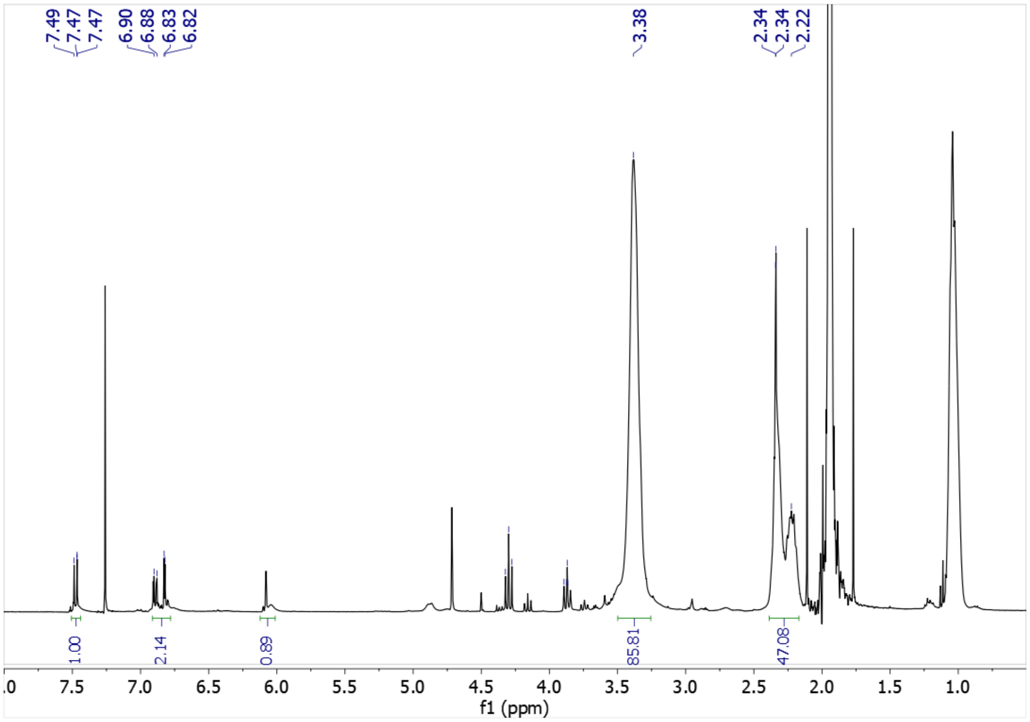

*Figure ESI 3. ^{1}H NMR spectrum of **PEtOx_Coum8** in $CDCl_3$*

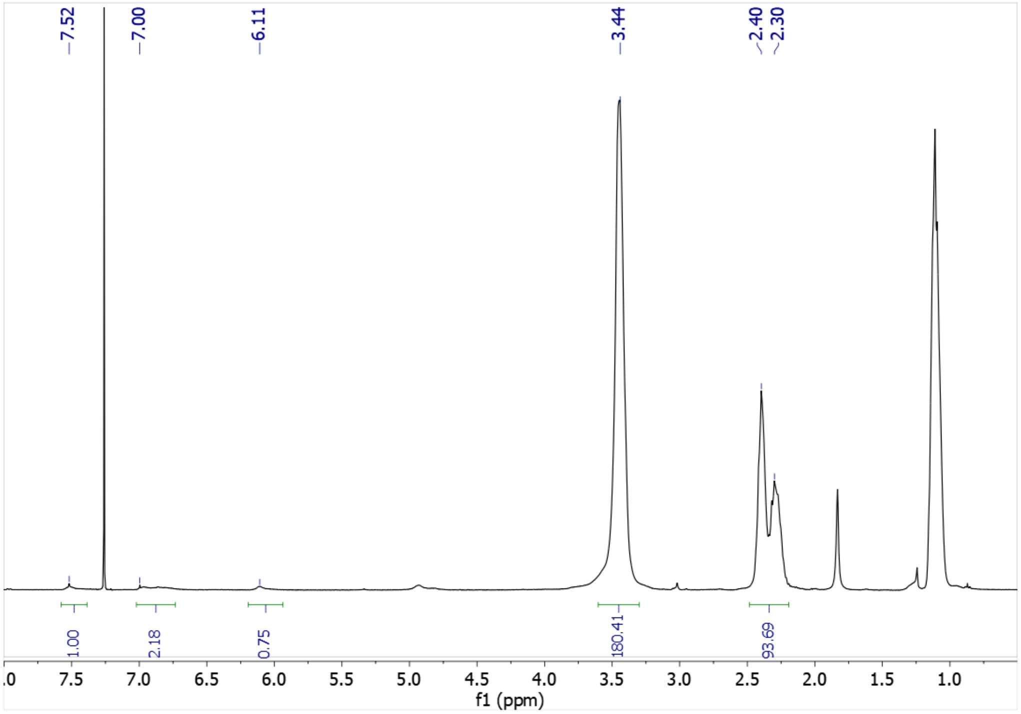

*Figure ESI 4. ^{1}H NMR spectrum of **PEtOx_Coum4** in $CDCl_3$*

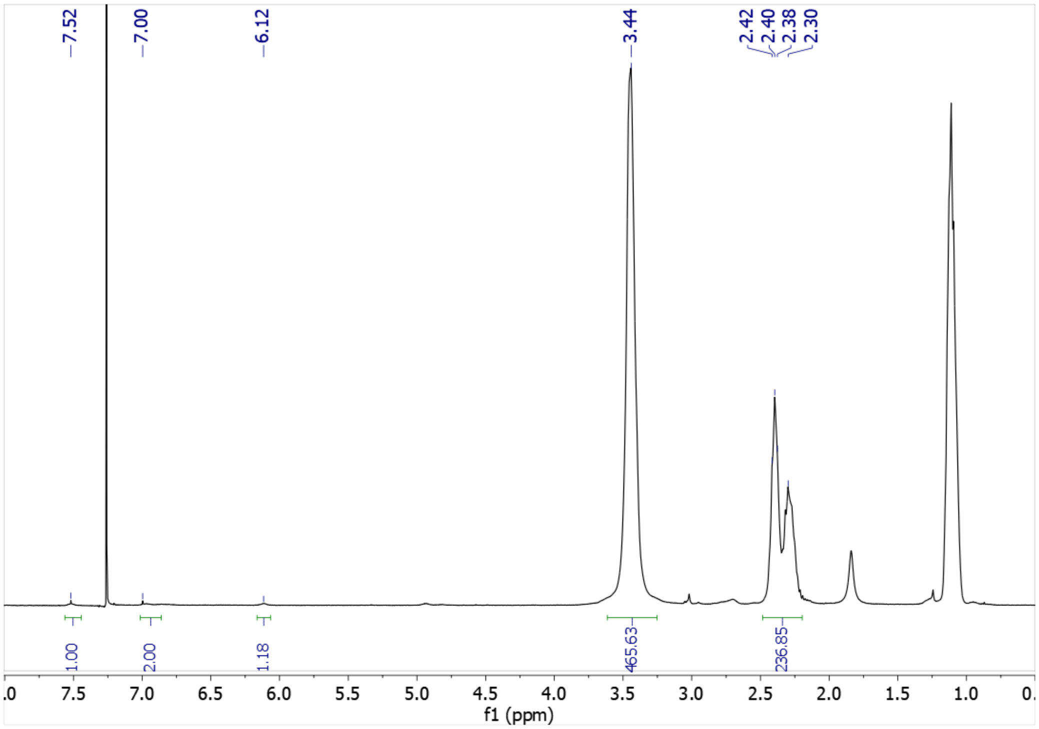

Figure ESI 5. [1]*H NMR spectrum of* ***PEtOx_Coum2*** *in* $CDCl_3$

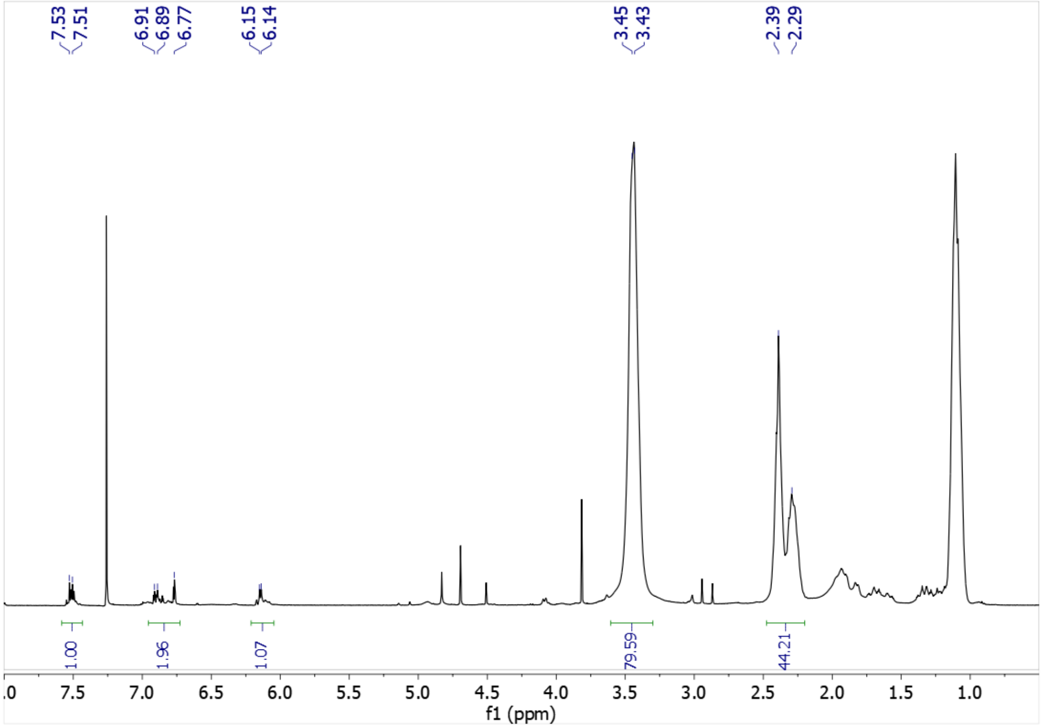

Figure ESI 6. [1]*H NMR spectrum of* ***PEtOx_Modif*** *in* $CDCl_3$

4 Crosslinking

The parameters used for all photorheology experiments are listed in Table ESI 2. Two drops of silicon oil were used to seal the gap between bottom plate and stamp of the rheometer to prevent the solvent from evaporating during the measurements.

Photorheological measurements were carried out with Anton Paar Modular Rheometer MCR 302 WESP. The detailed set-up description is presented in the work of Gorsche *et al*. [46]. Plate-to-plate configuration with the diameter 25 mm was used. Bottom immobile glass plate enables light irradiation. As the light source

For irradiation, an OmniCure LX400 UV-LED-spot curing system was used with a 365 nm LED. In order to measure the light intensity an Ocean Optics 2000+ USB device was used with the SpectraSuit. The polychromatic light source Omnicure type S2000-XLA with the range of wavelengths 320 – 500 nm and a single-tube liquid filled light guide with a diameter of 8 mm was also used in combination with photorheology. It was calibrated by EXFO R2000 Radiometer.

Table ESI 2. Rheology parameters used for photorheology experiments

Gap size	0.05 mm
Frequency f	1 Hz
Strain γ	0.02 %

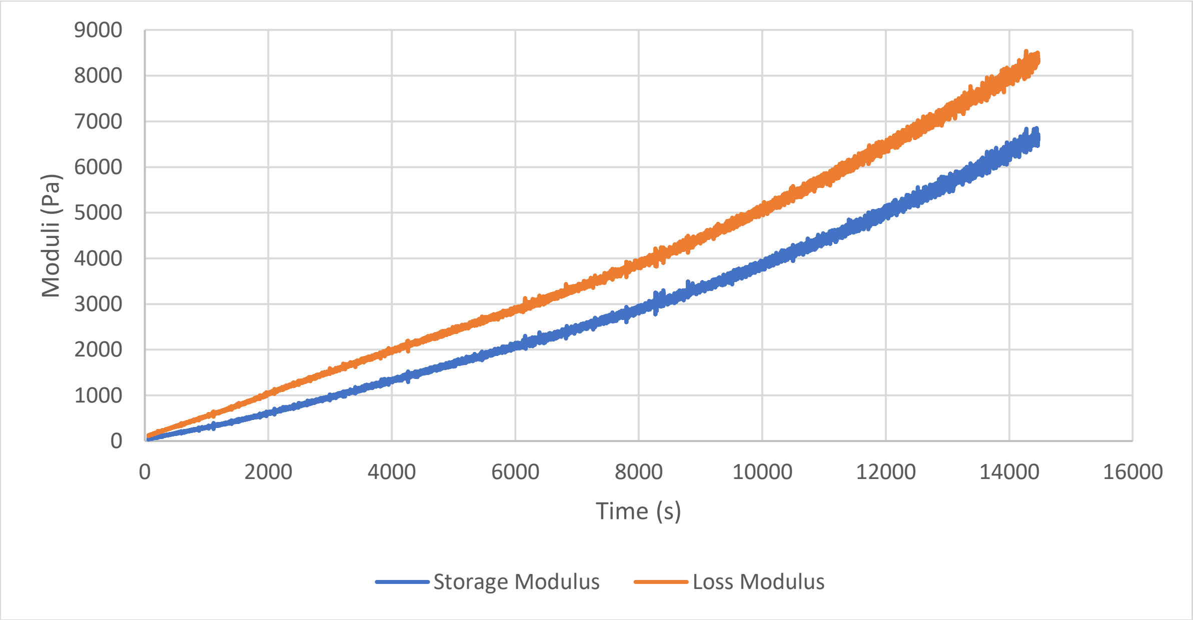

*Figure ESI 7. Photorheology measurement of 25 wt% **PEtOx_modif** in 1,4-dioxane irradiated with a monochromatic light source (365 nm, 15 mW·cm^{-2}).*

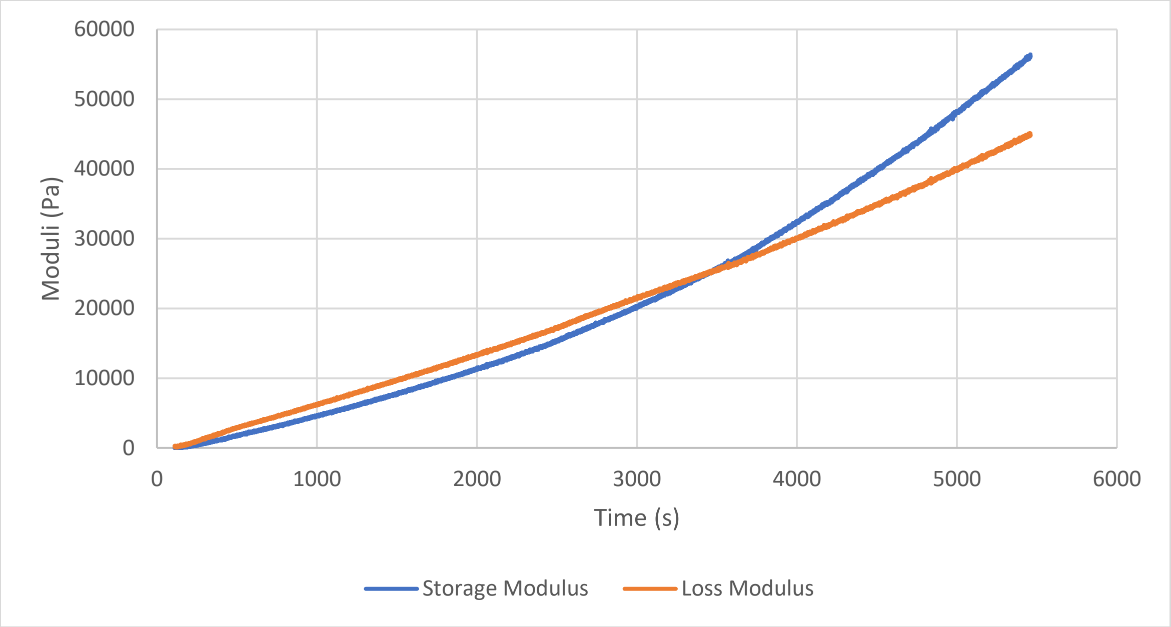

Figure ESI 8. Photorheology measurement of 25 wt% PEtOx_modif in 1,4-dioxane irradiated with a polychromatic light source (320 – 500 nm, 139 mW·cm-2).

Unfortunately, no gelation was observed in the conducted experiment using an LED as light source. Although we did observe a gel point on the Figure ESI 9 by using a polychromatic light source with higher intensity, we consider this to be a measurement artifact due to the drying of the sample at very long irradiation times.

5 Hydrogel formation and swelling studies

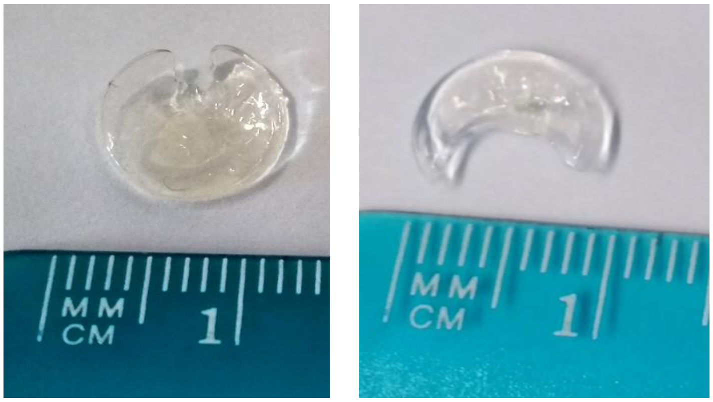

PEtOx_Coum8/PEGDA PEGDA

Figure ESI 9. Photographs of ***PEtOx_Coum8****/PEGDA hydrogel and pure PEGDA hydrogel after irradiation and swelling in distilled water.*

Table ESI 3. Gel content and swelling degree of crosslinked samples

Sample	*Gel content / %*	*Swelling degree / -*
PEtOx_Coum8/PEGDA	62.7±16.5	5.1±0.8
PEtOx_Coum4/PEGDA	52.2±11.6	7.1±0.9
PEtOx_Coum2/PEGDA	41.0±3.3	11.6±0.3
PEtOx_modif/PEGDA	70.4*	4.0*
PEGDA	86.1±8.0	2.5±0.6
PEtOx_modif	18.1±4.4	24.5±4.1
PEtOx_Coum8	13.4±1.3	16±3.5

* experiment performed in duplicate

6 Literature

[1] Dargaville TR, Park J-R, Hoogenboom R, (2018), Macromolecular Bioscience 18, 1800070.

[2] Kelly AM, Wiesbrock F, (2012), Macromol. Rapid Commun. 33, 1632-1647.

[3] Highley CB, Rodell CB, Burdick JA, (2015), Adv. Mater. 27, 5075-5079.

[4] https://gestis.dguv.de/data?name=490110, 14.07.2022.

[5] Nagata M, Yamamoto Y, (2008), React. Funct. Polym. 68, 915-921.

[6] Chujo Y, Sada K, Saegusa T, (1990), Macromolecules 23, 2693-2697.

[7] Kabb CP, O'Bryan CS, Deng CC, Angelini TE, Sumerlin BS, (2018), ACS Applied Materials & Interfaces 10, 16793-16801.

[8] Zahoranová A, Kroneková Z, Zahoran M, Chorvát Jr D, Janigová I, Kronek J, (2016), J. Polym. Sci., Part A: Polym. Chem. 54, 1548-1559.

[9] Viegas TX, Bentley MD, Harris JM, Fang Z, Yoon K, Dizman B, Weimer R, Mero A, Pasut G, Veronese FM, (2011), Bioconjugate Chem. 22, 976-986.

[10] Luxenhofer R, Schulz A, Roques C, Li S, Bronich TK, Batrakova EV, Jordan R, Kabanov AV, (2010), Biomaterials 31, 4972-4979.

[11] Leiske MN, Lai M, Amarasena T, Davis TP, Thurecht KJ, Kent SJ, Kempe K, (2021), Biomaterials 274, 120843.

[12] Pizzi D, Mahmoud AM, Klein T, Morrow JP, Humphries J, Houston ZH, Fletcher NL, Bell CA, Thurecht KJ, Kempe K, (2021), Eur. Polym. J. 151, 110447.

[13] Finnegan JR, Pilkington EH, Alt K, Rahim MA, Kent SJ, Davis TP, Kempe K, (2021), Chemical Science 12, 7350-7360.

[14] Hsiue G-H, Chiang H-Z, Wang C-H, Juang T-M, (2006), Bioconjugate Chem. 17, 781-786.

[15] Mero A, Pasut G, Via LD, Fijten MWM, Schubert US, Hoogenboom R, Veronese FM, (2008), J. Controlled Release 125, 87-95.

[16] Moreadith RW, Viegas TX, Bentley MD, Harris JM, Fang Z, Yoon K, Dizman B, Weimer R, Rae BP, Li X, Rader C, Standaert D, Olanow W, (2017), Eur. Polym. J. 88, 524-552.

[17] https://clinicaltrials.gov/ct2/show/NCT02579473, 08.12.2021.

[18] Seeliger W, Aufderhaar E, Diepers W, Feinauer R, Nehring R, Thier W, Hellmann H, (1966), Angewandte Chemie International Edition in English 5, 875-888.

[19] Kagiya T, Narisawa S, Maeda T, Fukui K, (1966), Journal of Polymer Science Part B: Polymer Letters 4, 441-445.

[20] Tomalia DA, Sheetz DP, (1966), Journal of Polymer Science Part A-1: Polymer Chemistry 4, 2253-2265.

[21] Bassiri TG, Levy A, Litt M, (1967), Journal of Polymer Science Part B: Polymer Letters 5, 871-879.

[22] Nahm D, (2021) PhD thesis, Julius-Maximilians-Universität Würzburg (Würzburg).

[23] Korchia L, Bouilhac C, Lapinte V, Travelet C, Borsali R, Robin J-J, (2015), Polymer Chemistry 6, 6029-6039.

[24] Breunig M, Lungwitz U, Liebl R, Fontanari C, Klar J, Kurtz A, Blunk T, Goepferich A, (2005), The Journal of Gene Medicine 7, 1287-1298.

[25] Aoi K, Okada M, (1996), Prog. Polym. Sci. 21, 151-208.

[26] Cesana S, Kurek A, Baur MA, Auernheimer J, Nuyken O, (2007), Macromol. Rapid Commun. 28, 608-615.

[27] Verbraeken B, Monnery BD, Lava K, Hoogenboom R, (2017), Eur. Polym. J. 88, 451-469.

[28] Bouten PJM, Hertsen D, Vergaelen M, Monnery BD, Catak S, van Hest JCM, Van Speybroeck V, Hoogenboom R, (2015), J. Polym. Sci., Part A: Polym. Chem. 53, 2649-2661.

[29] Hoogenboom R, Fijten MWM, Schubert US, (2004), J. Polym. Sci., Part A: Polym. Chem. 42, 1830-1840.

[30] Datta S, Jutková A, Šrámková P, Lenkavská L, Huntošová V, Chorvát D, Miškovský P, Jancura D, Kronek J, (2018), Biomacromolecules 19, 2459-2471.

[31] Weber C, Hoogenboom R, Schubert US, (2012), Prog. Polym. Sci. 37, 686-714.

[32] Oleszko-Torbus N, Utrata-Wesołek A, Wałach W, Dworak A, (2017), Eur. Polym. J. 88, 613-622.

[33] Hoogenboom R, Thijs HML, Jochems MJHC, van Lankvelt BM, Fijten MWM, Schubert US, (2008), Chem. Commun., 5758-5760.

[34] Hijazi M, Schmidt M, Xia H, Storkmann J, Plothe R, Santos DD, Bednarzick U, Krumm C, Tiller JC, (2019), Polymer 175, 294-301.

[35] Majerčíková M, Nádaždy P, Chorvát D, Satrapinskyy L, Valentová H, Kroneková Z, Šiffalovič P, Kronek J, Zahoranová A, (2021), Polymers 13.

[36] Eng YJ, Xu J, Sugiarto S, Jonnalagadda US, Ang W, Lee JH-C, Kwan JJ, Nguyen TM, (2021), ACS Applied Polymer Materials 3, 4264-4274.

[37] de la Rosa VR, Bauwens E, Monnery BD, De Geest BG, Hoogenboom R, (2014), Polymer Chemistry 5, 4957-4964.

[38] Tait A, Fisher AL, Hartland T, Smart D, Glynne-Jones P, Hill M, Swindle EJ, Grossel M, Davies DE, (2015), Biomaterials 61, 26-32.

[39] Šrámková P, Zahoranová A, Kelar J, Kelar Tučeková Z, Stupavská M, Krumpolec R, Jurmanová J, Kováčik D, Černák M, (2020), Scientific Reports 10, 9478.

[40] Zhang N, Pompe T, Amin I, Luxenhofer R, Werner C, Jordan R, (2012), Macromolecular Bioscience 12, 926-936.

[41] Milonaki Y, Kaditi E, Pispas S, Demetzos C, (2012), J. Polym. Sci., Part A: Polym. Chem. 50, 1226-1237.

[42] Hoogenboom R, Thijs HML, Wouters D, Hoeppener S, Schubert US, (2008), Macromolecules 41, 1581-1583.

[43] Trinh Che L, Hiorth M, Hoogenboom R, Kjøniksen A-L, in Polymers, Vol. 12, 2020.

[44] Li T, Tang H, Wu P, (2015), Langmuir 31, 6870-6878.

[45] Zhang N, Luxenhofer R, Jordan R, (2012), Macromol. Chem. Phys. 213, 1963-1969.

[46] Gorsche C, Harikrishna R, Baudis S, Knaack P, Husar B, Laeuger J, Hoffmann H, Liska R, (2017), Anal. Chem. 89, 4958−4968
